# Human organoid model of pontocerebellar hypoplasia 2a recapitulates brain region-specific size differences

**DOI:** 10.1242/dmm.050740

**Published:** 2024-07-22

**Authors:** Theresa Kagermeier, Stefan Hauser, Kseniia Sarieva, Lucia Laugwitz, Samuel Groeschel, Wibke G. Janzarik, Zeynep Yentür, Katharina Becker, Ludger Schöls, Ingeborg Krägeloh-Mann, Simone Mayer

**Affiliations:** ^1^ Hertie Institute for Clinical Brain Research, University of Tübingen, 72076 Tübingen, Germany; ^2^ Graduate Training Centre of Neuroscience, University of Tübingen, 72076 Tübingen, Germany; ^3^ German Center for Neurodegenerative Diseases, 72076 Tübingen, Germany; ^4^ International Max Planck Research School, Graduate Training Centre of Neuroscience, University of Tübingen, 72076 Tübingen, Germany; ^5^ Department of Neuropediatrics, Developmental Neurology and Social Pediatrics, University of Tübingen, 72076 Tübingen, Germany; ^6^ Department of Neuropediatrics and Muscle Disorders, Center for Pediatrics and Adolescent Medicine, Medical Center, Faculty of Medicine, University of Freiburg, 79106 Freiburg, Germany; ^7^ Heidelberger Akademie der Wissenschaften, 69117 Heidelberg, Germany

**Keywords:** Organoid, PCH2a, Apoptosis, Cerebellum, Differentiation, Rare disease

## Abstract

Pontocerebellar hypoplasia type 2a (PCH2a) is an ultra-rare, autosomal recessive pediatric disorder with limited treatment options. Its anatomical hallmark is hypoplasia of the cerebellum and pons accompanied by progressive microcephaly. A homozygous founder variant in *TSEN54*, which encodes a tRNA splicing endonuclease (TSEN) complex subunit, is causal. The pathological mechanism of PCH2a remains unknown due to the lack of a model system. Therefore, we developed human models of PCH2a using regionalized neural organoids. We generated induced pluripotent stem cell (iPSC) lines from three males with genetically confirmed PCH2a and subsequently differentiated cerebellar and neocortical organoids. Mirroring clinical neuroimaging findings, PCH2a cerebellar organoids were reduced in size compared to controls starting early in differentiation. Neocortical PCH2a organoids demonstrated milder growth deficits. Although PCH2a cerebellar organoids did not upregulate apoptosis, their stem cell zones showed altered proliferation kinetics, with increased proliferation at day 30 and reduced proliferation at day 50 compared to controls. In summary, we generated a human model of PCH2a, providing the foundation for deciphering brain region-specific disease mechanisms. Our first analyses suggest a neurodevelopmental aspect of PCH2a.

## Teaser

**Summary:** A human regionalized neural organoid model for pontocerebellar hypoplasia, an ultra-rare neurological disorder, provides the foundation to decipher the brain region-specific disease mechanism *in vitro*.

## INTRODUCTION

Pontocerebellar hypoplasias (PCHs) are a heterogeneous group of neurogenetic disorders characterized by severe neurodevelopmental impairment and hypoplasia of the cerebellum and pons (van Dijk et al., 2018). Besides the primary pontocerebellar hypoplasia, affected individuals develop progressive microcephaly (Barth et al., 2007; Namavar et al., 2011). PCH2 is the most common form of PCH but it is still ultra-rare (estimated 1:100,000 births) (Budde et al., 2008; Sanchez-Albisua et al., 2014). Clinically, PCH2 is characterized by profound neurodevelopmental delay, causing a significant burden to affected individuals and their families (Ammann-Schnell et al., 2021; Sanchez-Albisua et al., 2014). To date, no curative treatment is available, and disease management is focused on alleviating symptoms.

Ninety percent of PCH2 cases are caused by a homozygous founder variant in the *TSEN54* gene (OMIM *608755; NM_207346.3:c.919G>T, p.Ala307Ser) and are referred to as PCH2a (OMIM #277470) (Budde et al., 2008; van Dijk et al., 2018). *TSEN54* encodes a subunit of the tRNA splicing endonuclease (TSEN) complex, which is involved in excising introns from a subset of pre-tRNAs by providing structural support and mediating substrate recognition (Chan and Lowe, 2009; Trotta et al., 2006; Yuan et al., 2023; Zhang et al., 2023). The TSEN54 protein region that contains the Ala307Ser variant is not visualized by single-particle cryogenic electron microscopy, suggesting that it is unstructured (Hayne et al., 2023; Sekulovski et al., 2023). Although bi-allelic pathogenic variants in *TSEN54* do not affect the endonuclease activity of the TSEN complex in fibroblasts of affected individuals, they result in a thermal destabilization of the complex and altered tRNA pools (Sekulovski et al., 2021). The TSEN54 protein is expressed throughout the body at varying levels (Human Protein Atlas) (Uhlen et al., 2015). At the mRNA level, *TSEN54* is widely expressed in the developing human brain (Human Brain Transcriptome) (Kang et al., 2011) starting in the first trimester of gestation (Kasher et al., 2011). In the second trimester of gestation, *TSEN54* is expressed highly in the developing cerebellum, pons and olivary nuclei (Budde et al., 2008). Its expression does not appear to be cell type-specific in the developing neocortex and cerebellum at the level of mRNA (Aldinger et al., 2021; Nowakowski et al., 2017). Collectively, from the TSEN54 expression pattern alone, it is unclear why specifically the cerebellum and pons are affected in PCH2a (Kasher et al., 2011). Interestingly, additional variants in *TSEN54* (associated with PCH4 and PCH5), as well as variants in other subunits of the TSEN complex (associated with PCH2b, PCH2c and PCH2f) and variants in *CLP1* (associated with PCH10), which encodes a protein that interacts with the TSEN complex, result in clinical phenotypes related to PCH2a (Schaffer et al., 2019). Therefore, it has been hypothesized that specific brain areas are especially vulnerable to TSEN malfunction due to a specific requirement of this complex during perinatal and early postnatal development (Budde et al., 2008).

Histopathological analysis to date has revealed a reduced complexity of cerebellar foliation, patches of Bergmann glia, loss of Purkinje cells (PCs), and severely reduced and misplaced granule cells (GCs), the major neuronal cell types of the cerebellar cortex (Rudnik-Schöneborn et al., 2014). Efforts to recapitulate PCH2a in animal models have not determined the cellular mechanism of pathophysiology so far (Schmidt et al., 2022; Kasher et al., 2011). Although the TSEN complex is conserved from archaea, *TSEN54* has undergone evolutionary changes in the primate lineage (Lee et al., 2016). Notably, the amino acid sequence around *TSEN54*:c.919G>T (p.Ala307Ser) is not conserved between species (Budde et al., 2008). Of the frequently used model organisms, only mouse and chicken share the amino acid residue (Budde et al., 2008). A recently developed fly model of PCH shows defects in brain development, death at larval stages and apoptosis upon loss of function of the *TSEN54* ortholog, but the relevance to the brain region-specific clinical phenotype is unclear (Schmidt et al., 2022). In zebrafish, loss of *tsen54* function leads to cell death in the brain during development (Kasher et al., 2011). A complete loss of *Tsen54* in mice results in early embryonic lethality (Ermakova et al., 2018). Moreover, a bi-allelic missense variant of *TSEN54* in dogs leads to a neurological disorder characterized by leukodystrophy, a disease with a strikingly different pathophysiology (Stork et al., 2019). These findings imply a potential species-specific effect of TSEN54 malfunction on brain development. Additionally, the brain has changed tremendously in evolution, especially in the primate lineage (Herculano-Houzel, 2012). Compared to the mouse neocortex, the human neocortex has expanded dramatically (1000× in the number of neurons and surface area), has a protracted development (neurogenesis is 20× longer than in mouse) and displays an unprecedented cellular heterogeneity (Geschwind and Rakic, 2013; Miller et al., 2019). Similarly, the human cerebellum has a 750-fold greater surface area than that of the mouse cerebellum, a protracted development occurring over 2-3 years compared to 30-35 days in mice, and additional transient stem cell zones that cannot be found in mice (Haldipur et al., 2022). Taken together, the lack of appropriate models of PCH2a has to date precluded the elucidation of its cellular and molecular pathology.

As an alternative to animal models, human brain organoid models have recently been increasingly used to model neurodevelopmental and early-onset neurodegenerative disorders as well as investigating environmental impacts on brain development (Eichmüller and Knoblich, 2022; Sarieva and Mayer, 2021). In order to study brain region-specific biology, it is possible to guide the differentiation of organoids to a specific brain region by adding specific morphogens (Fleck et al., 2021; Kadoshima et al., 2013; Sloan et al., 2018). Neocortical organoids, for instance, recapitulate cell type composition of the developing human neocortex with SOX2^+^ ventricular radial glia cells organized into rosettes reminiscent of the ventricular zone (VZ) and shed other neural progenitor cells (NPCs) and neurons to putative subventricular zone and cortical plate regions, respectively (Pasca et al., 2015). Within the cortical plate-like regions, a distinct lamination pattern emerges, featuring CTIP2 (BCL11B)^+^ deep-layer and SATB2^+^ upper-layer excitatory neurons (Pasca et al., 2015).

Similarly, in cerebellar organoid differentiation protocols (Muguruma et al., 2015; Silva et al., 2020), the stem cells of two cerebellar NPC zones are established: KIRREL2^+^/PTF1A^+^ VZ and BARHL1^+^/ATOH1^+^ rhombic lip (RL) (Leto et al., 2016; Mizuhara et al., 2010). These NPCs generate neurons of two major cerebellar neuronal identities: PCs and GCs, respectively. PC precursors (KIRREL2^+^/PTF1A^+^) are derived from the VZ and identified by OLIG2/SKOR2 expression, and subsequently mature into calbindin (CALB or CALB1)^+^ PCs. (Leto et al., 2016; Wang et al., 2011). RL-derived precursors (BARHL1^+^/ATOH1^+^) differentiate into GCs and glutamatergic deep cerebellar nuclei neurons, which are positive for LHX2 (Ballabio et al., 2020; Kamei et al., 2023; Lago et al., 2023; Morales and Hatten, 2006).

**Fig. 1.**
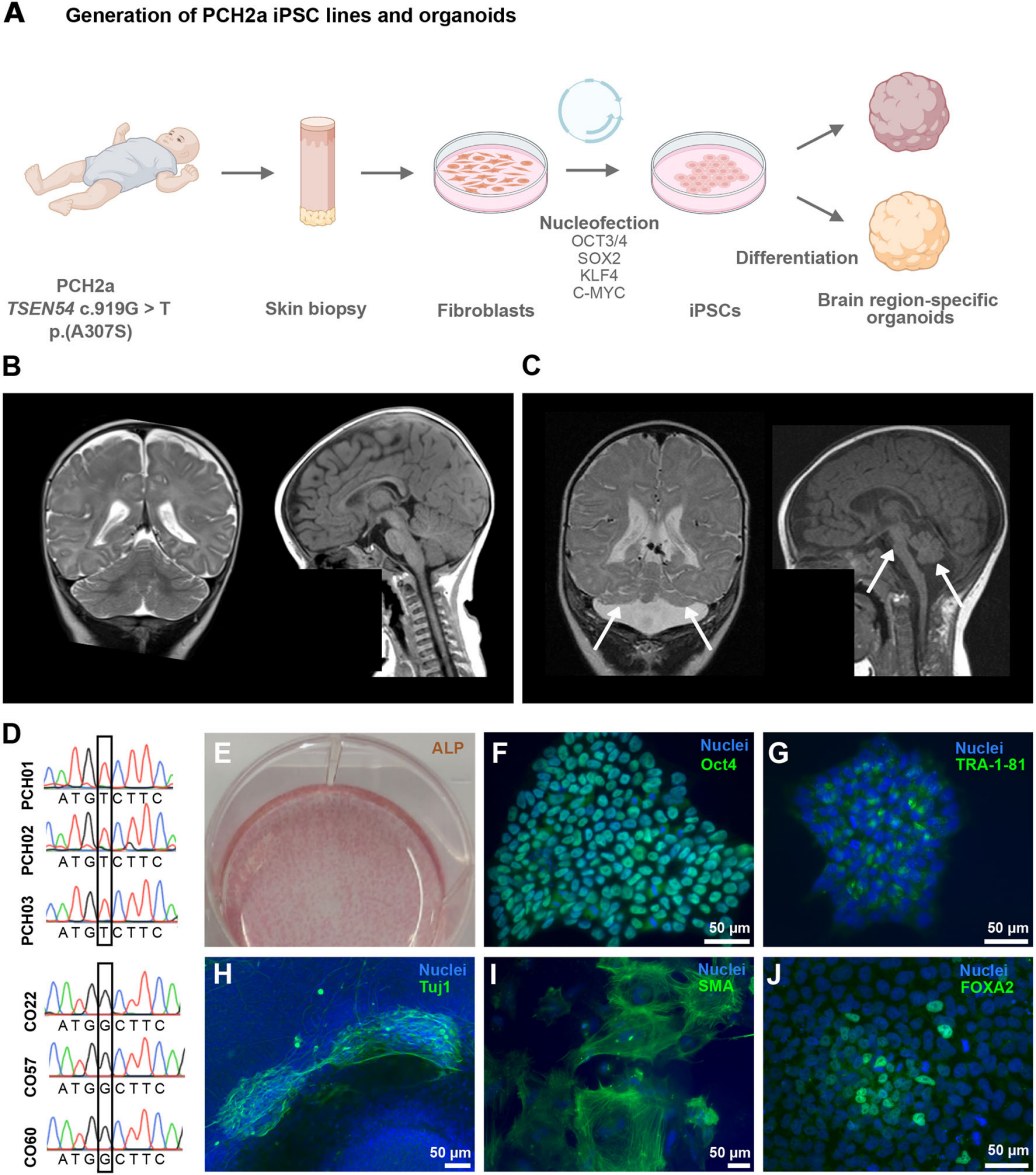
**Generation of PCH2a iPSCs.** (A) Experimental scheme of PCH2a induced pluripotent stem cell (iPSC) generation (scheme was created with BioRender.com). (B) Magnetic resonance imaging (MRI) scans of the brain of a normally developing infant (6 months of age) and (C) an infant with PCH2a (6 months of age, donor of iPSC line PCH02). T2-weighted coronal images (B,C, left) show cerebellar hemispheres severely reduced in size (indicated by arrows) in the PCH2a child compared to the control individual. T1-weighted sagittal images (B,C, right) illustrate the severe pontine and cerebellar hypoplasia in the PCH2a child (indicated by arrows). The facial regions of the MRI scans are covered in order to protect the privacy of both individuals. (D) Sanger sequencing results of PCH2a iPSCs verifies the *TSEN54* c.919G>T point mutation, whereas control lines show the c.919G genotype (homozygous). (E) Alkaline phosphatase (ALP) staining of undifferentiated iPSCs. (F,G) Immunocytochemical staining of undifferentiated iPSCs for the pluripotency markers OCT4 (F) and TRA-1-81 (G) and DAPI (nuclei) demonstrates the pluripotency of generated cell lines. (H-J) Immunocytochemical staining of iPSCs spontaneously differentiated into the three germ layers illustrates the differentiation potential of the generated iPSCs. Cells were stained for Tuj1 (tubulin βIII marker, ectoderm) (H), smooth muscle actin (SMA, mesoderm) (I) and forkhead box A2 (FOXA2, endoderm) (J), as well as DAPI (nuclei). Representative images show iPSCs from the cell line PCH01.

In this study, we leveraged the recent developments in the generation of regionalized neural organoids (Zhang et al., 2022) to create a human model of PCH2a. We identified three individuals that display the genetic, clinical and brain imaging features previously described for PCH2a. We then derived induced pluripotent stem cells (iPSCs) from donated fibroblasts (Fig. 1A) from these three affected children and three healthy individuals (all male, Table 1). We did not find differences in proliferation and cell death between PCH2a and control iPSCs. Next, we differentiated PCH2a and control iPSCs towards a cerebellar and neocortical fate in three-dimensional (3D) cultures. Growth curves of organoids recapitulated the brain region-specific pathology observed in affected individuals. Cerebellar PCH2a organoids were severely reduced in size compared to controls starting early in differentiation, whereas neocortical PCH2a organoids showed less severe divergence from controls only at later stages of differentiation. We did not detect differences in the induction of apoptosis in NPCs. Instead, we found an increased number and thickness of neural rosettes along with increased proliferation in these areas in early but not late differentiation stages in PCH2a cerebellar organoids compared to controls. Our study thus provides first insights into the disease mechanism, which can be analyzed in depth in future studies by using the *in vitro* models of PCH2a established here.

**Table 1.**
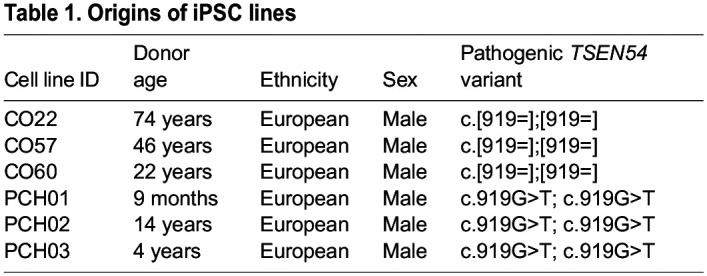
Origins of iPSC lines

## RESULTS

### Generation of PCH2a-derived iPSCs

It is unclear how a ubiquitously expressed gene involved in tRNA metabolism causes pathology only in specific tissues and even within the nervous system, with differential pathology in different brain regions. Based on recent successes in modeling neurogenetic disorders in organoids (Khakipoor et al., 2020; Velasco et al., 2020), we reasoned that a human iPSC-based regionalized neural organoid model could recapitulate pathological hallmarks of PCH2a (Fig. 1A). Previously, cerebellar organoids were used to investigate the mechanisms of medulloblastoma, a pediatric cerebellar cancer (Ballabio et al., 2020; Lago et al., 2023). We hypothesized that generating neocortical organoids and cerebellar organoids might model the brain region-specific pathology in PCH2a. To our knowledge, cerebellar organoids have not yet been used to model neurogenetic disorders involving the cerebellum.

As a first step, we recruited three affected males (Fig. 1B,C) with genetically confirmed PCH2a, harboring the c.919G>T variant in the *TSEN54* gene in the homozygous state (Fig. 1D). Clinical features typical of PCH2a (Sanchez-Albisua et al., 2014) were evident in all probands (Table S1). In summary, subjects displayed severe developmental delay with minimal cognitive and motor development, seizures of variable semiology and a severe dystonic movement disorder. Moreover, the probands exhibited dysphagia and gastrointestinal disturbances (Table S1). Neuroimaging revealed severe hypoplasia of the brainstem, pons and cerebellum in contrast to less severe volume reduction of the cerebrum, as seen in an affected child (donor of iPSC line PCH02) (Fig. 1C) in comparison to an age-matched control (Fig. 1B).

We obtained skin biopsies from these three probands, extracted fibroblasts and derived iPSCs using an episomal reprogramming approach (Fig. 1A) (Okita et al., 2013). iPSCs were subjected to a range of quality controls. First, pluripotency was confirmed through staining for alkaline phosphatase (ALP) (Fig. 1E) and immunocytochemistry for OCT4 and the TRA-1-81 antigen podocalyxin (PODXL) (Fig. 1F,G). The differentiation potential was corroborated using spontaneous tri-lineage differentiation (Korneck et al., 2022). iPSCs differentiated into the three primordial germ layers, namely, the ectoderm (marked by the Tuj1 antibody against tubulin βIII or TUBB3), the mesoderm (identified by an antibody against smooth muscle actin or SMA) and the endoderm (identified by anti-FOXA2) (Fig. 1H-J). Control lines used in this study were generated following the same protocol and were subjected to all aforementioned quality controls (see Materials and Methods for a detailed description). Single nucleotide polymorphism (SNP) array analysis of all six lines used in this study revealed no larger chromosomal aberrations induced by the reprogramming (Data S1). Additionally, control lines did not harbor the c.919G>T variant (Fig. 1D).

**Fig. 2.**
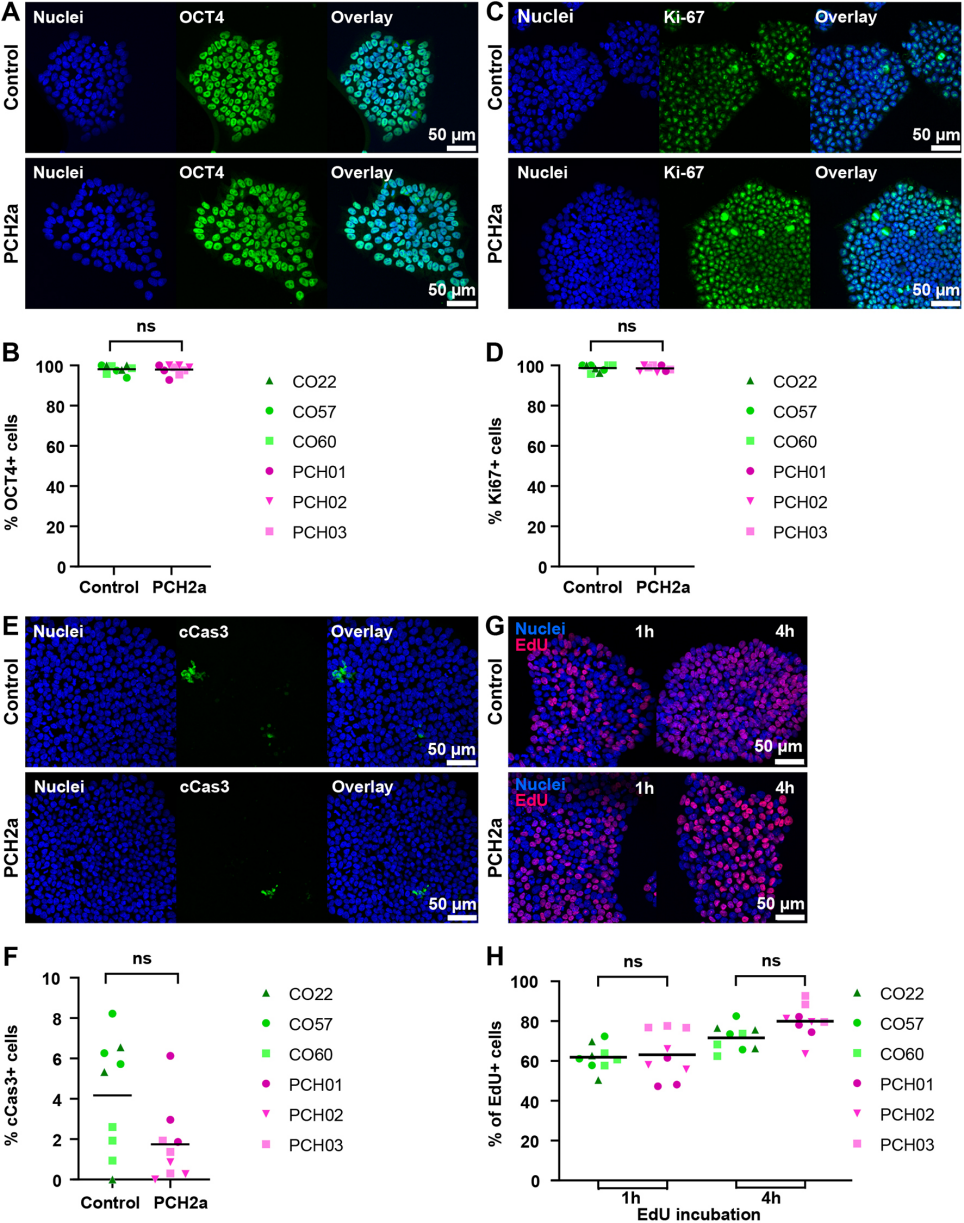
**PCH2a-derived and control iPSCs do not differ in expression of pluripotency, proliferation and apoptosis markers.** (A,C) Immunocytochemical staining of PCH2a and control iPSCs for the pluripotency marker OCT4 (A) and the proliferation marker Ki67 (C) confirmed their expression in all iPSC lines [representative images are of iPSCs from the cell lines CO22, passage (P) 19, and PCH01, P17 (A), and CO57, P20, and PCH03, P20 (B)]. (B,D) Quantification of OCT4^+^ cells (B) and Ki67^+^ cells (D) normalized to DAPI-based cell count showed no significant difference in the number of OCT4^+^ or Ki67^+^ cells between PCH2a and control iPSCs (assessment of three passages per cell line). (E) Immunocytochemical staining of PCH2a and control iPSCs for the apoptosis marker cCas3 showed low expression levels in all iPSC lines (representative images are of iPSCs from CO60, P18, and PCH02, P18). (F) Quantification of cCas3^+^ cells, normalized to DAPI-based cell count showed no significant difference in number of cCas3^+^ cells between PCH2a and control iPSCs (assessment of three passages per cell line). (G) Visualization of cell proliferation by click-chemistry detection of EdU in PCH2a and control iPSCs after 1 and 4 h of incubation with EdU (representative images are of iPSCs from CO60, P18, and PCH02, P18). (H) Quantification of EdU-positive cells, normalized to DAPI-based cell count after 1 and 4 h of EdU incubation showed no significant difference in the number of EdU-positive cells between PCH2a and control iPSCs (assessment of three passages per cell line). ns, *P*>0.05 [two-tailed unpaired *t*-test with Welch's correction assuming unequal standard deviations (SDs)].

In order to determine even subtle differences in iPSC properties between PCH2a and control lines, we assessed and quantified the number of cells expressing the pluripotency marker OCT4 (Fig. 2A,B), the proliferative marker Ki67 (MKI67) (Fig. 2C,D) and the apoptotic marker cleaved caspase-3 (cCas3; encoded by *CASP3*) (Fig. 2E,F) of three consecutive passages of all PCH2a and control iPSC lines used in this study. We identified no significant differences in the number of cells expressing these markers between PCH2a and control lines. Additionally, we incubated the iPSC lines with 5-ethynyl-2ʹ-deoxyuridine (EdU) for 1 and 4 h to assess the proliferation rate (Fig. 2G,H). Quantification of EdU^+^ cells revealed no significant difference in EdU incorporation between PCH2a and control lines. In conclusion, the pathogenic variant does not affect the proliferation and viability of the iPSCs. indicating that iPSCs derived from affected individuals maintain characteristics of control iPSC lines and can be used for studying tissue-specific pathology by employing regionalized neural organoid differentiation protocols.

### Human brain organoids have brain region-specific growth deficits

**Fig. 3.**
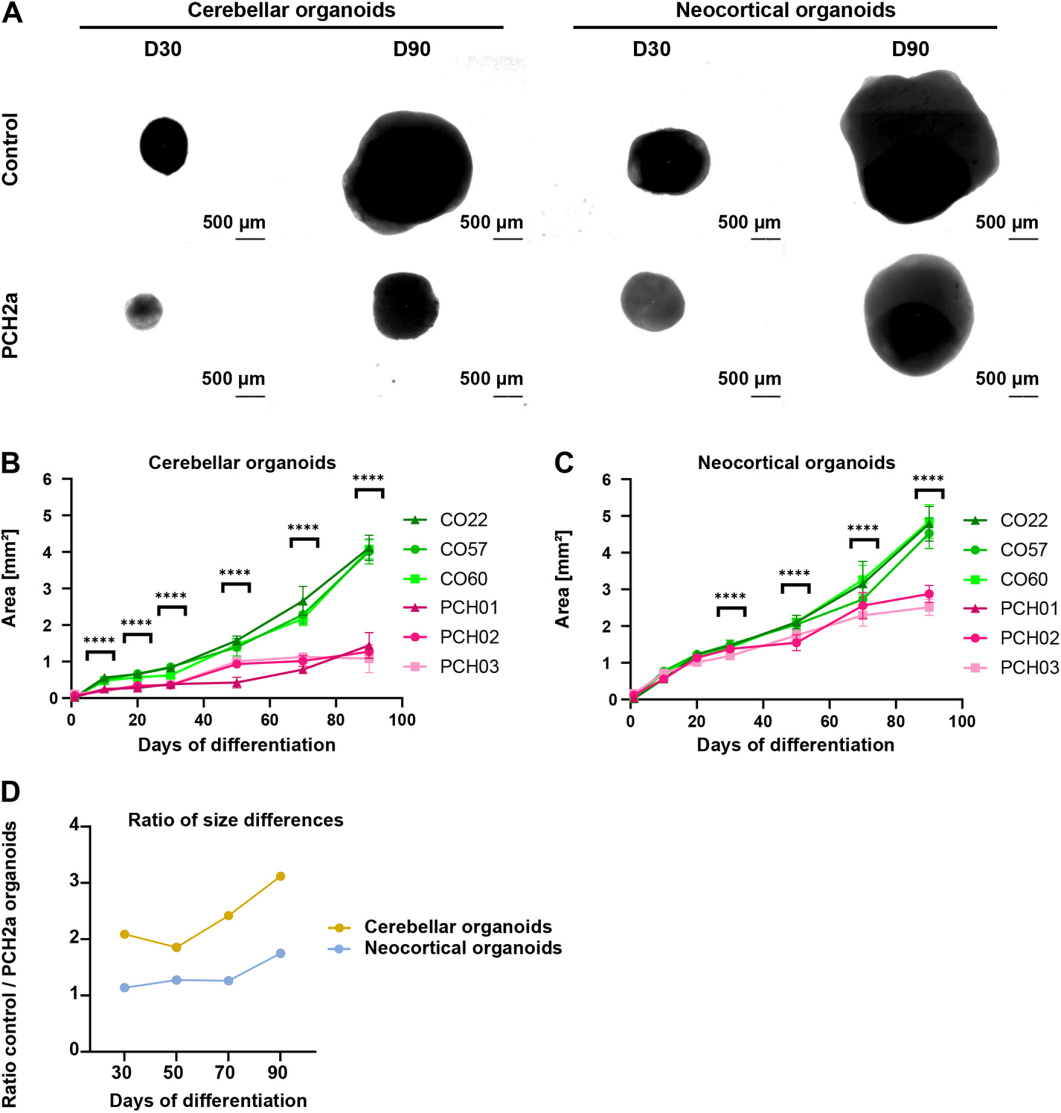
**PCH2a organoids are significantly smaller than control organoids.** (A) Representative brightfield images of cerebellar and neocortical organoids in culture at day (D) 30 and D90 of differentiation illustrate the differences in size. (B,C) Growth curves of PCH2a and control cerebellar (B) and neocortical (C) organoids, differentiated from three different cell lines per condition (derived from three different individuals), show the area of the organoids in the images pictured in A during the culture period of 90 days. Cerebellar organoids (B) differed significantly in size from D10 of differentiation, with discrepancies increasing over time. Neocortical organoids (C) showed significant differences from D30 of differentiation. *n*>8 organoids per cell line, timepoint and differentiation. Note that PCH01 neocortical differentiation is absent due to a contamination. Points represent the mean, error bars represent s.e.m. ****P<0.05 (two-tailed unpaired *t*-test with Welch's correction assuming unequal SDs). (D) The ratio between mean sizes of control and PCH2a organoids calculated from data presented in B,C. The ratios increased over time and were higher within cerebellar differentiation.

To model brain region-specific pathology, we then differentiated three control and the three PCH2a iPSC lines into cerebellar (Silva et al., 2020) and neocortical organoids (Pasca et al., 2015) (formally referred to as cortical spheroids; Pasca et al., 2022) using established protocols (Fig. 3A).

We first determined whether cerebellar hypoplasia and progressive microcephaly found in affected individuals (Fig. 1B,C) were recapitulated in our *in vitro* model (Fig. 3B,C). We therefore measured the sizes of both cerebellar and neocortical organoids in brightfield images at day (D) 10, D20, D30, D50, D70 and D90. During neocortical differentiation, one PCH2a line was contaminated and the differentiation was terminated at D20. Interestingly, for both brain region-specific differentiations, we found significant differences in size between PCH2a and control organoids (Fig. 3A-C; Fig. S1A-F). On average, cerebellar PCH2a organoids were smaller than controls starting from D10 of differentiation (Fig. 3B), whereas neocortical PCH2a organoids showed differences from D30 onwards (Fig. 3C). Statistical assessment (three-way ANOVA) revealed no significant difference for the sizes within the two experimental groups (PCH2a or control) at any time point, justifying the comparison of mean sizes between groups. Linear regression models were significantly different between control and PCH2a organoids of both regional identities (Fig. S1G,H, Table S2). At D50, the ratio between the mean sizes of control and PCH2a cerebellar organoids was 1.85, increasing to 3.12 at D90 (Fig. 3D). In neocortical organoids, the ratio between the mean sizes of control and PCH2a organoids was 1.13 at D50 and 1.74 at D90 of differentiation (Fig. 3D). The regionalized neural organoid growth curves resembled brain morphometry in PCH2a-affected individuals with early detection of cerebellar hypoplasia in the first months of life (Sanchez-Albisua et al., 2014) and later progressive microcephaly (Ekert et al., 2016), albeit at a different time scale.

### PCH2a-derived iPSCs differentiate towards cerebellar and neocortical fate

**Fig. 4.**
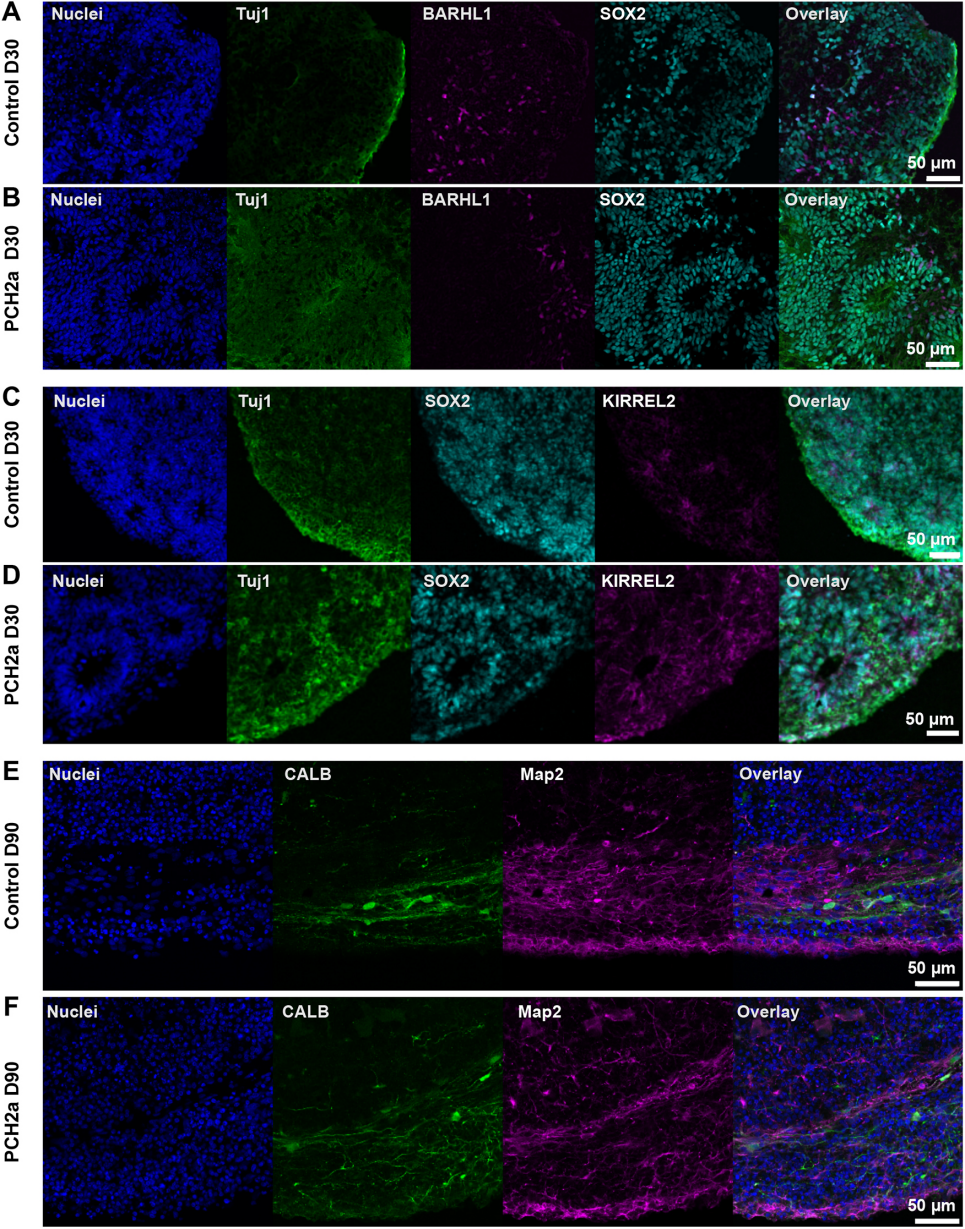
**PCH2a and control cerebellar organoids show differentiation into the cerebellar lineage.** Immunohistochemistry of control and PCH2a cerebellar organoid sections at D30 and D90 showed differentiation into the cerebellar lineage. (A,B) Expression of the early neuronal marker Tuj1 (green), the neural precursor marker SOX2 (cyan) and the glutamatergic precursor marker BARHL1 (magenta) in D30 control (A) and PCH2a (B) cerebellar organoids (representative images show organoids derived from CO22, P19, and PCH02, P17). (C,D) The GABA-ergic precursor marker KIRREL2 (magenta) is expressed in D30 cerebellar control (C) and PCH2a (D) organoids together with Tuj1 (green) and SOX2 (cyan) (representative images are of iPSCs from CO22, P19, and PCH01, P19). (E,F) Control (E) and PCH2a (I) cerebellar organoids show calbindin (CALB, cyan) in postmitotic Purkinje cells and neuronal marker MAP2 (magenta) expression (representative images are of iPSCs from CO57, P18, and PCH03, P19).

To start assessing the cellular underpinnings of the brain region-specific growth deficits (Fig. 3), we analyzed the presence of different progenitor and neuronal cell types in cerebellar and neocortical organoids at different time points in PCH2a and control organoids. We found robust neural differentiation in both PCH2a and control cerebellar organoids based on immunohistochemical analysis for the markers SOX2 (NPCs) and Tuj1 (immature neurons) at D30 (Fig. 4A,B). Moreover, cerebellar organoids demonstrated the presence of both RL- and VZ-derived cerebellar NPC populations. Immunohistochemistry against BARHL1 (Fig. 4A,B) and ATOH1 (Fig. S2A-D) confirmed the presence of RL-derived glutamatergic cerebellar precursor cells. Cerebellar VZ derivates such as GABAergic cerebellar precursors are characterized by KIRREL2 (Fig. 4C,D), OLIG2 (Fig. S2E,F) and SKOR2 (Fig. S3A-D) in immunohistochemistry at D30. At later stages of differentiation (D90), we found CALB- and MAP2-positive cells, indicating the presence of PCs in cerebellar organoids (Fig. 4E,F). Similarly, in neocortical organoids, immunohistochemical analysis for CTIP2, SATB2, SOX2 and Tuj1 collectively revealed neocortical differentiation and generation of layer-specific excitatory neurons (Fig. S3A-F). We found expression of the NPC marker SOX2 in neural rosettes surrounded by CTIP2-positive deep-layer excitatory neurons at D50 in control (Fig. S3A) and PCH2a (Fig. S3B) neocortical organoids. In addition, expression of the upper-layer excitatory neuron marker SATB2 was found at D70 and D90 (Fig. S3C-F). Taken together, the acquisition of regionalized neural fate and neuronal maturation was evident in both cerebellar and neocortical control and PCH2a organoids, suggesting that they can be used to analyze molecular and cellular changes induced by the disease-causing variant.

### Expression of the apoptotic marker cCas3 is not altered in PCH2a cerebellar and neocortical organoids

**Fig. 5.**
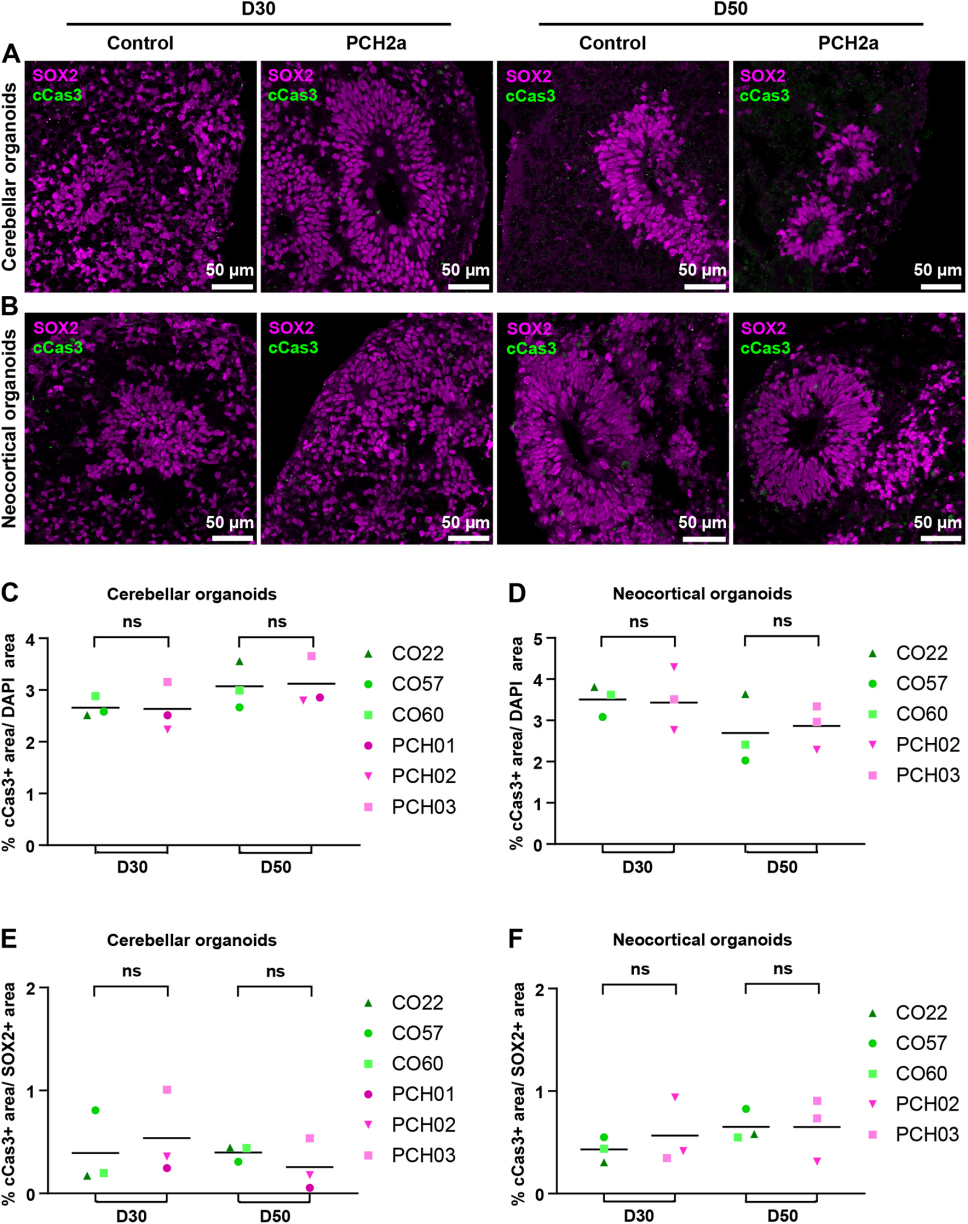
**Expression of the apoptotic marker cCas3 is not altered in PCH2a organoids.** (A,B) Confocal microscopy images of immunohistochemistry on cerebellar (A) and neocortical (B) organoid sections at D30 and D50 of differentiation show expression of the neural precursor marker SOX2 and the apoptotic marker cCas3 in rosette-like structures of organoids. Representative images of cerebellar organoids (A) are from CO22, P19, and PCH03, P19 (D30/D50), and those for neocortical organoids (B) are from CO57, P18, and PCH02, P17 (D30/50). (C,D) Quantification of the cCas3-positive area over DAPI signal showed no significant difference in cCas3 expression between PCH2a and control in cerebellar (C) and neocortical (D) organoids at D30 and D50 of differentiation. (E,F) Quantification of cCas3-positive area within the SOX2^+^ area showed no significant difference in cCas3 expression between PCH2a and control in cerebellar (E) and neocortical (F) organoids at D30 and D50 of differentiation. ns, not significant, *P*>0.05 (two-tailed unpaired *t*-test with Welch's correction assuming unequal SDs).

Previous histopathological assessment of a PCH2a-affected individual suggested a degenerative nature of the disease (Barth et al., 2007; Rudnik-Schöneborn et al., 2014). Additionally, studies on zebrafish and fruit fly *TSEN54* ortholog loss-of-function models indicated that hypoplasia resulted from cell death (Kasher et al., 2011; Schmidt et al., 2022). We therefore investigated whether elevated levels of apoptosis could explain the reduced size of PCH2a cerebellar and neocortical organoids. However, immunohistochemistry for the apoptotic marker cCas3 did not reveal differences between control and PCH2a cerebellar (Fig. 5A) and neocortical (Fig. 5B) organoids. Quantitative analysis of the cCas3-positive area over the DAPI-positive area of individual regions of interest (ROIs) (SOX2-rich regions containing SOX2^+^ rosettes and surrounding cells) did not show a significant difference between PCH2a and control organoids at D30 and D50 in both cerebellar (Fig. 5C) and neocortical (Fig. 5D) organoids. Further quantification of the cCas3^+^ area within the SOX2^+^ area only did not show elevated apoptosis in the SOX2^+^ NPC population either (Fig. 5E,F). Taken together, we observed a significant size difference in PCH2a brain organoids (Fig. 3) in the absence of obvious changes in apoptosis at D30 and D50 of differentiation in cerebellar and neocortical organoids (Fig. 5).

### PCH2a cerebellar organoids show earlier establishment of dense SOX2^+^ structures

**Fig. 6.**
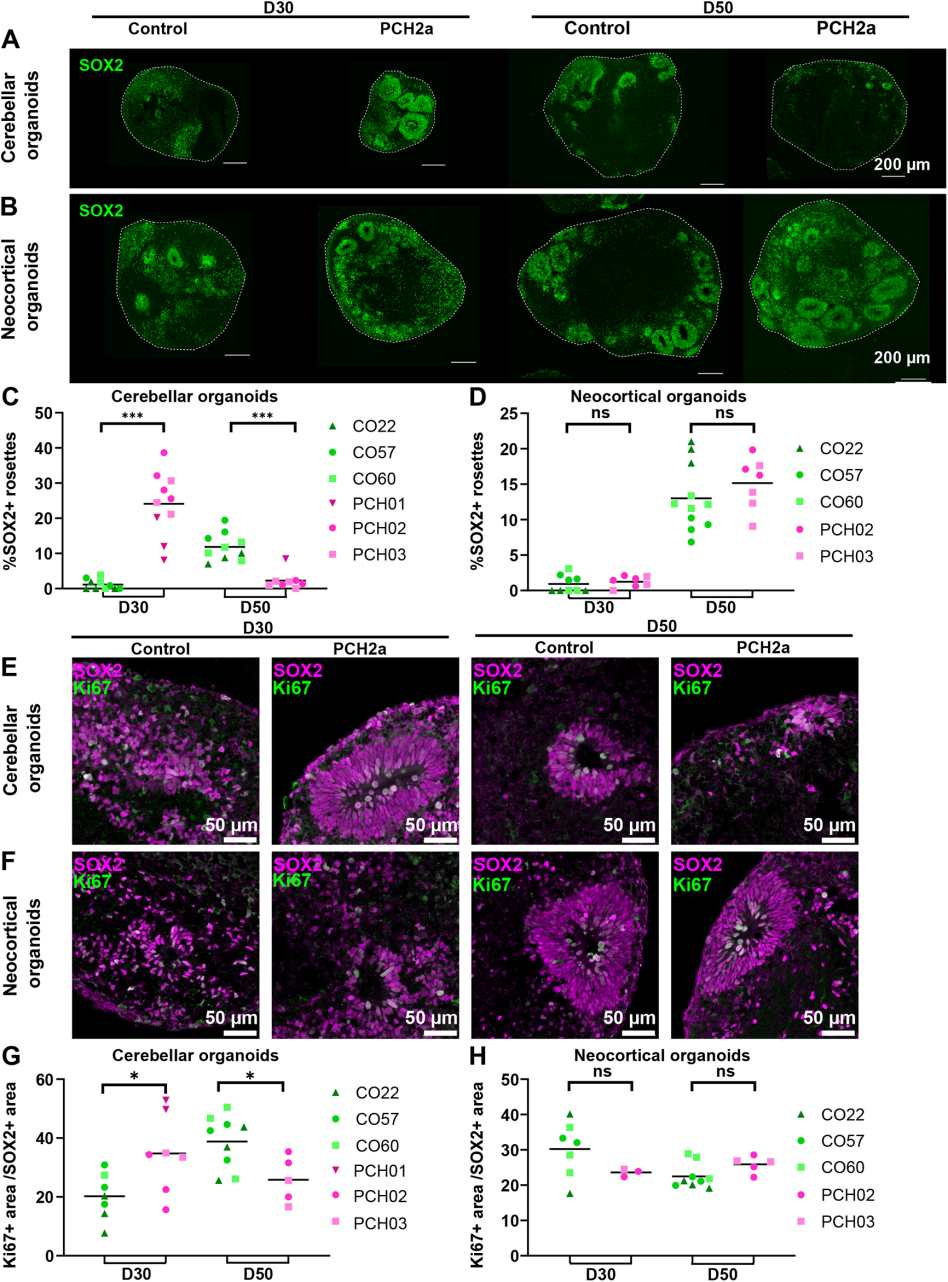
**PCH2a cerebellar organoids show earlier establishment of dense SOX2^+^ structures, whereas neocortical organoids demonstrate no difference in SOX2^+^ structures.** (A,B) Epifluorescence images of immunohistochemistry on cerebellar (A) and neocortical (B) organoids at D30 and D50 of differentiation show the expression of SOX2 (NPCs) in control (left) and PCH2a (right) organoids. Representative images of cerebellar organoids (A) are from CO57, P18, and PCH02, P17 (D30); and CO57, P18, and PCH03, P19 (D50); those for neocortical organoids (B) are from CO57, P18, and PCH02 P17 (D30); and CO22, P19, and PCH02, P17 (D50). (C,D) Quantitative analysis of the area covered by SOX2^+^ structures normalized to the area of the organoid. (C) PCH2a cerebellar organoids showed a significantly higher proportion of dense SOX2^+^ structures at D30, whereas control organoids showed these structures at D50. (D) Neocortical organoids did not show significant differences at D30 and D50. (E,F) Confocal images of immunohistochemistry against Ki67 (magenta) and Tuj1 (green) in cerebellar (E) and neocortical (F) organoid sections at D50 illustrate the expression of Ki67 in PCH2a (right) and control (left) organoids within SOX2^+^ structures. Representative images for cerebellar organoids (E) are from CO57, P18, and PCH03, P19 (D30); and CO57, P18, and PCH03, P19 (D50); and those for neocortical organoids (F) are from CO60, P19, and PCH02, P17 (D30); and CO60, P19, and PCH02, P17 (D50). The PCH2a cerebellar organoid at D30 is also used to illustrate quantification in Fig. S5A-C. (G,H) Quantitative analysis of percentage of Ki67^+^ area normalized to SOX2^+^ area in cerebellar and neocortical organoids at D30 and D50 of differentiation. (G) PCH2a cerebellar organoids showed a significantly higher proportion of Ki67^+^ area at D30. This difference was reversed at D50 of differentiation. (H) Neocortical organoids did not demonstrate significant differences at D30 and D50 of differentiation in the Ki67^+^/SOX2^+^ area. ns, not significant, *P*>0.05; *P<0.05; ***P<0.001 (two-tailed unpaired *t*-test with Welch's correction assuming unequal SDs).

Instead of apoptosis, an alternative hypothesis that could explain the reduced size of cerebellar and neocortical organoids is altered proliferation of NPCs. We therefore assessed possible changes in SOX2^+^ NPCs through immunohistochemistry. Analysis at D30 and D50 of differentiation showed that the number and structure of SOX2^+^-rich rosette structures differed between PCH2a and control cerebellar organoids (Fig. 6A) with no apparent differences in neocortical organoids (Fig. 6B). In D30 PCH2a cerebellar organoids, SOX2^+^ rosettes took up 24±3.07% (mean±s.e.m.) of the total organoid area, whereas in control, only 2±0.53% of the organoid area was taken up by SOX2^+^ rosettes (Fig. 6C). At D50, this difference was reversed: control cerebellar organoids showed a larger area of SOX2^+^ structures over the total organoid area (12±1.24%) than PCH2a organoids (2±0.92%). In contrast, neocortical organoids did not show differences in the area of SOX2^+^ rosette structures over the total organoid area on both D30 and D50 of differentiation (Fig. 6D). As they differentiated from D30 to D50, the percentage of SOX2^+^ rosettes increased in both PCH2a and control neocortical organoids (Fig. 6D).

Different factors such as morphogen gradients or viral infections can influence the morphology of SOX2^+^ structures in brain organoids (Albanese et al., 2020; Pagliaro et al., 2023). To determine whether SOX2^+^ rosette structures also possessed a distinct morphology in cerebellar PCH2a organoids, we quantified the average size and thickness of the SOX2^+^ structures. At D30 of differentiation, PCH2a cerebellar organoids had significantly bigger and thicker SOX2^+^ structures than those of controls; this difference was reversed at D50 (Fig. S4A,C). Neocortical PCH2a organoids did not show significant differences in the size of SOX2^+^ structures at D30 or D50 of differentiation compared to controls (Fig. S4B). There was a slight reduction in the thickness of SOX2^+^ structures in PCH2a neocortical organoids compared to those in controls at D30 (Fig. S4D), but not at D50 (Fig. S4D). To investigate whether the altered SOX2^+^ rosette structures in cerebellar and neocortical organoids also translated to higher proliferation within these structures, we quantified the proportion of Ki67^+^ cells among SOX2^+^ cells (Fig. 6E,F). We found higher proliferation of the SOX2^+^ cells (Fig. 6G) in D30 PCH2a cerebellar organoids compared to that in controls. Conversely, the proliferation of SOX2^+^ cells was lower in D50 PCH2a cerebellar organoids compared to that in controls (Fig. 6G). We did not find any differences in the proportion of Ki67^+^/SOX2^+^ cells between PCH2a and control neocortical organoids (Fig. 6H). Taken together, these analyses indicate aberrant proliferation properties of the NPCs in cerebellar organoids. In line with the severity of the growth deficit (Fig. 3), dramatic changes in progenitor cell properties were seen in PCH2a cerebellar organoids compared to controls, whereas only subtle differences were found in neocortical organoids when comparing PCH2a and control differentiations. Taken together, we propose that size differences between PCH2a and control lines in cerebellar and neocortical organoids are explained predominantly by proliferation differences rather than increased apoptosis.

## DISCUSSION

Understanding the cellular and molecular mechanisms of PCH2a has been hampered to date by the lack of a model replicating neuroanatomical hallmarks of the disorder such as the brain region-specific hypoplasia. Moreover, no study to date has modeled the specific variant underlying PCH2a in a neural cellular context. In this study, we aimed to close this gap by generating human brain region-specific organoid models of PCH2a. To achieve that, we (1) derived three iPSC lines from affected individuals (Fig. 1), (2) compared these lines with three control iPSC lines generated by the same protocol (Figs 1 and 2), and (3) extensively characterized PCH2a and control iPSC-derived cerebellar and neocortical organoids (Figs 3–6) to start elucidating the mechanisms underlying cerebellar hypoplasia and microcephaly. We found that, although PCH2a-derived iPSCs did not differ from control lines (Fig. 2), both cerebellar and neocortical organoids demonstrated disease-relevant phenotypes. Thus, we propose that human regionalized brain organoids can serve as models to study cellular and molecular mechanisms underlying PCH2a in a tissue-specific manner.

### Using patient-derived iPSCs to model rare diseases

An important consideration in light of the current study is the suitability of using patient-derived iPSCs for disease modeling *in vitro*. A major concern in this context is the reproducibility between iPSC lines derived from non-related individuals with distinct genetic backgrounds. It has been reported that the major source of variation between different iPSC lines is their genetic background (Volpato and Webber, 2020). Genetic background inevitably differs between PCH2a-derived and control cell lines and thus serves as a technical confounder that may increase variation (Volpato and Webber, 2020). It may also allow overlooking additional genetic variants that affect the disease course but were missed in the causal gene identification. In order to mitigate such confounding effects, we analyzed iPSC properties in detail (Fig. 2) and found no differences between PCH2a and control lines. In our organoid differentiations, we also achieved robust differences between the PCH2a and control lines despite the genetic differences. Importantly, PCH2a is a genetically homogenous disorder, characterized by a single homozygous missense mutation, and may thus be particularly suited to such an experimental design. Furthermore, we did not find significant differences in organoid sizes within the control or PCH2a group (Fig. 3). The fact that we can generate highly reproducible data while using cell lines derived from unrelated individuals can also be seen as a strength of our study. The lack of genetic engineering using CRISPR-Cas9 also means that no off-target effects can confound our results. In conclusion, although we show consistent results between the three control lines and the three PCH2a lines, respectively, an important extension of our study will be to confirm our findings in isogenic cell lines.

### Brain region-specific anatomical hallmarks recapitulated in organoids

In individuals affected by PCH2a, a cerebellum reduced in size is already present at birth, whereas a progressive reduction of cerebral volumes may be detected with time, suggesting an ongoing atrophic process of neurodegeneration (Ekert et al., 2016). Analogously, we found that cerebellar PCH2a organoids were severely reduced in size from early stages on, whereas neocortical PCH2a organoids started displaying differences in growth at later stages of development (Fig. 3). Additionally, differences in cerebellar organoid size between PCH2a and controls at later stages were larger compared to those for neocortical PCH2a and control organoids (Fig. 3). In patients, the atrophy of supratentorial structures could be either caused by a primary effect of the disease-causing variant or a consequence of the lack of inputs from the cerebellum, as has been discussed in studies of very preterm infants with cerebellar lesions (Limperopoulos et al., 2014). Our data on the reduced size of neocortical organoids starting at D30 of differentiation indicate that the disease-causing *TSEN54* variant directly affects neocortical development. We therefore suggest that primary as well as secondary effects of the pathogenic variant on cerebellar and neocortical development may contribute to the diverse clinical phenotype of PCH2a. Notably, just 20% of the human cerebellum is involved in motor function (Haldipur et al., 2022; Marek et al., 2018), and cerebellar hypoplasia disrupting cerebellar–cerebral projections may, thus, also contribute directly to the pathological hallmarks of PCH2a not related to motor function, such as neurodevelopmental delay and lack of language development.

### Novel insights into the disease mechanism of PCH2a using the organoid models

Our model provides the foundation for studying the cellular and molecular disease mechanisms underlying PCH2a as we can generate species-specific biomaterial with regionalized neural fate for subsequent analysis of the biochemical and cellular differences. Elucidating the mechanisms of PCH2a has implications for PCH subtypes that are caused by variants in TSEN genes and *CLP1* (Schaffer et al., 2019). Furthermore, it can further be relevant for other rare neurological disorders caused by defects in the tRNA-processing machinery (Schaffer et al., 2019).

Mutations in *TSEN54* lead to aberrant tRNA pools in human fibroblasts (Sekulovski et al., 2021). Assuming that aberrant tRNA pools are also present in the cerebellar and neocortical PCH2a organoids generated in this study, it remains elusive how they translate to the reduced size in PCH2a organoids resembling the clinical phenotype of affected individuals. Interestingly, in different model systems, tRNAs can directly regulate apoptosis (Avcilar-Kucukgoze and Kashina, 2020; Mei et al., 2010). Apoptosis has been suggested to occur in the cerebellum of affected individuals with PCH based on neuropathological observations in infants, children and adults (Barth et al., 2007) as well as in animal models of PCH (Kasher et al., 2011; Schmidt et al., 2022). We therefore investigated whether elevated apoptosis in our cerebellar and neocortical PCH2a organoids may explain the observed size differences (Fig. 5). At the very early time points of differentiation when neural progenitor cells are the dominant cell population, which were the focus of this study, we did not find any differences in apoptosis between PCH2a and control cerebellar and neocortical organoids (Fig. 5). It is possible that at later stages of differentiation, as neurons mature, apoptosis occurs, recapitulating human pathology. Future work addressing apoptosis rates in later stages of cerebellar organoid differentiation, which have recently been optimized and shown to generate more mature neuronal populations such as functional PCs (Atamian et al., 2024; Chen et al., 2023), will be informative to answer this question.

Alternatively, altered tRNA pools could directly affect differentiation. Specific tRNAs can change cell state and regulate proliferation and differentiation in concert with mRNAs (Gingold et al., 2014). Interestingly, in cancer, specific tRNAs can even promote metastatic progression (Goodarzi et al., 2016). We therefore hypothesize that the developing cerebellum and, to a lesser extent, the developing neocortex have specific requirements for appropriate tRNA pools throughout differentiation (Gao et al., 2024), originating perhaps from the increased neuronal output (Haldipur et al., 2022; Miller et al., 2019). It has been suggested that TSEN is required for processing cerebellum-specific pre-tRNAs (Sekulovski et al., 2021). Indeed, tRNA isodecoders display tissue-specific expression (Ishimura et al., 2014; Pinkard et al., 2020) and tRNA modifications change as oligodendrocyte precursor cells differentiate into oligodendrocytes (Martin et al., 2022), indicating that tRNA pools may be important regulators of neural lineage progression.

In this study, cerebellar organoids showed altered proliferation, indicated by an increased thickness and number of SOX2^+^ rosettes, area of SOX2^+^ rosettes over total organoid area (Fig. 6A-D), and percentage of Ki67^+^/SOX2^+^ dividing NPCs (Fig. 6E-H). Moreover, the direction of the changes in NPC proliferation was reversed from D30 to D50 of differentiation: PCH2a cerebellar organoids at D30 of differentiation appeared to consist largely of proliferative SOX2^+^ cells, whereas they lost the majority of SOX2^+^ area by D50. Such a change is seemingly counterintuitive to the smaller size of the PCH2a cerebellar organoids compared to that of control organoids over the course of differentiation. Although this phenomenon may be explained through an altered balance between proliferation and differentiation in the cerebellar organoids, the exact mechanism cannot be identified conclusively from our current study. Further in-depth analysis of our organoid model using, for instance, single-cell transcriptomics, as recently demonstrated on control cerebellar organoids (Atamian et al., 2024; Nayler et al., 2021), will reveal cellular and molecular differences between PCH2a and control organoids to explain the differences in size.

Based on our findings of altered proliferation in cerebellar organoids, we hypothesize that impaired proliferation and differentiation in the cerebellum leads to hypoplasia. Supporting this hypothesis, it has been reported that a different form of cerebellar hypoplasia (PCH17), caused by bi-allelic variants in *PRDM13* (OMIM *6167441 and #619909), comes with early disruption of cerebellar and brain stem development (Coolen et al., 2022). These neuropathological findings were supported by loss-of-function experiments in zebrafish, which revealed disruption of PC differentiation (Coolen et al., 2022). Moreover, altered development in several brain structures has been reported in human brain samples of a subtype of PCH (Patel et al., 2006). However, elucidating the exact mechanism of how defective TSEN54 function results in the clinical phenotypes of cerebellar hypoplasia and progressive microcephaly requires further mechanistic studies, and should ultimately be confirmed with pathological specimen and 3D reconstruction of magnetic resonance images of human brains at different developmental stages.

### Regionalized neural organoid models for neurogenetic disorders

Human brain organoid models have been used extensively to study neurogenetic disorders in a human cellular context (Khakipoor et al., 2020; Velasco et al., 2020). In most cases, cerebral organoid models have been used, even when the cerebellum was primarily affected by the disorder (Bras et al., 2022), as cerebellar differentiation protocols have been developed only recently (Atamian et al., 2024; Chen et al., 2023; Hua et al., 2022; Muguruma et al., 2015; Nayler et al., 2021; Silva et al., 2020). Therefore, to date, the use of cerebellar organoids in neurogenetic disease modeling has not been demonstrated. For the first time in this study, we show how cerebellar organoids can be used to model the brain region-specific neuropathology of PCH2a, a severe neurological disorder that primarily affects the cerebellum, pons and, to a lesser extent, the neocortex. PCH2a iPSCs robustly differentiated into both the neocortical and cerebellar fate (Figs 4 and 5), in line with the observation that regional specification was not affected in a *tsen54* loss-of-function zebrafish (Kasher et al., 2011). Moreover, evidence for different cerebellar cell types including PCs and GCs were found in pathological studies of PCH2a (Rudnik-Schöneborn et al., 2014), indicating functional neural tube regionalization. However, we observed brain region-specific differences in organoid growth and proliferation between PCH2a and control organoids. We therefore suggest that using 3D differentiation protocols that replicate regional identity of the affected brain region is crucial to study a specific neuropathology. Consequently, if a disease affects multiple brain regions, the full potential of organoid technology may only be leveraged by combining different brain region-specific organoids.

## MATERIALS AND METHODS

### Recruitment of affected individuals

Affected individuals were recruited within our PCH2 natural history study, collecting clinical and diagnostic data, including diagnostic magnetic resonance images. Written informed consent was obtained from guardians and archived. All procedures were performed in accordance with the Helsinki Declaration. Individual-level data were deidentified. The study was approved by the ethics committee of the medical faculty, the local Institutional Review Boards of the Medical Faculty of the University of Tübingen, Germany (961/2020BO2 and 598/2011BO1), and Freiburg, Germany (20-1040).

### Diagnostic confirmation by genetic sequencing

Next-generation sequencing and/or Sanger sequencing was performed after obtaining written informed consent for either clinical sequencing and/or center-specific institutional review board-approved research sequencing. All affected individuals harbored the hypomorphic founder variant c.919G>T in *TSEN54* in the homozygous state. The bi-allelic localization was confirmed by carrier testing.

### Skin biopsies

Skin biopsies were acquired at different ages (9 months to 15 years) according to local standards of routine diagnostic procedures.

### Culturing and reprogramming fibroblasts

Human dermal fibroblasts were obtained from skin biopsies and cultivated in Dulbecco's modified eagle medium (DMEM; Thermo Fisher Scientific) supplemented with 10% fetal bovine serum (FBS; Thermo Fisher Scientific) (fibroblast medium).

iPSC generation from fibroblasts was performed according to a published protocol with minor modifications (Okita et al., 2013). Briefly, reprogramming was initiated by nucleofection of 1×10^5^ fibroblast with 1 µg of each episomal plasmid [pCXLE-hUL (Addgene #27080), pCXLE-hSK (Addgene #27078) and pCXLE-hOCT4 (Addgene #27076)] using the Nucleofector 2b transfection device (Lonza). Initially, fibroblasts were cultivated in fibroblast medium supplemented with 2 ng/ml FGF2 (Peprotech, 100-18B). On D3, the medium was changed to Essential 8 (E8) medium [DMEM/F12 (Thermo Fisher Scientific, 31330095), 64 mg/l L-ascorbic acid 2-phosphate magnesium (Sigma-Aldrich, A8960), 1% insulin-transferrin-selenium-supplement (100×) (Thermo Fisher Scientific, 41400045), 10 ng/ml FGF2 (Peprotech, 100-18B), 2 ng/ml TGFβ1 (Peprotech, 100-21C) and 100 ng/ml heparin (Sigma-Aldrich, H3393)] containing 100 µM sodium butyrate (NaB; Sigma-Aldrich, B5887). After 3-4 weeks, with medium changes every other day, iPSC colonies were manually picked and further expanded in E8 medium, performing medium changes daily. After ≥5 passages, they were genomically and functionally characterized and frozen in E8 medium containing 40% KO-SR (Thermo Fisher Scientific, 10828-028), 10% DMSO (Sigma-Aldrich, D4540) and 1 µM Y-27632 (Selleck Chemicals, S1049). All iPSC lines used in this study (three control lines and three PCH2a lines) were characterized according to the scientific guidelines for Lab Resources (https://www.sciencedirect.com/journal/stem-cell-research/about/lab-resources#scientific-guidelines-for-lab-resources).

### Genomic integrity analysis

In order to verify genomic integrity, DNA of iPSCs and fibroblasts was isolated with DNeasy Blood and Tissue Kit (QIAGEN) according to the manufacturer's guidelines. Whole-genome SNP genotyping (SNP array) was conducted using Infinium OmniExpressExome-8-BeadChip (Illumina) and GenomeStudio V2.0.3 (Illumina) for evaluation. Copy number analysis was performed using the CNVPartition plugin (Illumina). Early mosaicism states were evaluated by manual review of B allele frequency plots on a chromosomal level. Results can be found in Data S1.

### Pluripotency assessment

To assess ALP expression or for immunocytochemical analysis, iPSCs were fixed with 4% paraformaldehyde (PFA; Morphisto, 11762) and either assessed for ALP expression or permeabilized with 0.1% Triton X-100 (Sigma-Aldrich, T8787), blocked with 5% FBS and stained overnight at 4°C with primary antibodies for immunocytochemical analysis (rabbit anti-OCT4, 1:100, Proteintech, 11263-1-AP; and mouse anti-TRA-1-81, 1:500, Millipore, MAB4381). Samples were visualized after staining with Alexa Fluor 488-conjugated secondary antibodies (goat anti-rabbit and goat anti-mouse IgGs, 1:1000, Invitrogen, A-11001 and A-11008) for 1 h at room temperature. Nuclei were counterstained with Hoechst 33342 (1:10,000, Invitrogen). Samples were embedded in ProLong Gold Antifade Reagent (Thermo Fisher Scientific, P36930) and imaged with AxioImager Z1 (Zeiss).

The differentiation capacity of iPSCs into cells of all three germ layers was determined by an embryonic body (EB)-based protocol. 1.2×10^6^ iPSCs were seeded in AggreWell800 plates (STEMCELL Technologies) in EB medium consisting of DMEM/F-12 (Gibco, 31330095) supplemented with 20% Knockout Serum Replacement (Gibco, 10828028), 1% MEM non-essential-amino-acid solution (Sigma-Aldrich, M5550-100ML), 1% penicillin/streptomycin (Sigma-Aldrich, P0781), 1% GlutaMAX (Thermo Fisher Scientific, 35050038) and 50 µM β-mercaptoethanol. On D4, EBs were plated onto coverslips for further differentiation. Specific expression of the markers TUJ (mouse anti-TUJ, 1:1000, Sigma-Aldrich, T8660) and SMA (mouse anti-SMA, 1:100, Dako, M0851) was assessed after 10 days as described above. For endodermal induction of iPSCs, 2×10^5^ cells were seeded onto coverslips and cultivated in endoderm induction medium consisting of RPMI1640 Advanced (Gibco, 12633012) supplemented with 1× B27 (Thermo Fisher Scientific, 17504044), 1% penicillin/streptomycin, 0.2% fetal calf serum (FCS; Gibco, A5256701), 2 µM CHIR-99021 (Tocris, 4423) and 50 ng/ml activin A (Peprotech, 120-14P). At D4 of differentiation, cells were stained for FOXA2 (rabbit anti-FOXA2, 1:300, Millipore, 07-633), following the fixation and immunocytochemistry protocol mentioned above.

### Sanger sequencing

To ensure the correct genetic background of all iPSC lines and control, Sanger sequencing was performed. Genomic DNA was extracted using the DNA Isolate kit (BioCat, BIO-52066-BL). The region of interest was amplified using the Phusion High-Fidelity PCR Kit (New England Biolabs, E0553S). The PCR product was purified with QIAquick PCR Purification Kit (QIAGEN, 28104) and samples were sent to Eurofins for sequencing. The following primers were used: forward, 5ʹ-AGAAACCCCAGGAGT-3ʹ, and reverse, 5ʹ-CTCAATCCATCCGAG-3ʹ.

### iPSC culture

iPSC lines derived from affected individuals and control lines were generated following the same protocol and cultured under standard conditions (37°C, 5% CO_2_ and 100% humidity) in E8 Flex medium (Gibco, A2858501) on human embryonic stem cell-qualified growth factor-reduced Matrigel-coated (Corning, 354277) cell culture dishes (Greiner, 657160). Passaging was performed in colonies using Gentle Dissociation Reagent (STEMCELL Technologies, 07174) once the culture reached 80-90% confluency. The culture medium was supplemented with thiazovivin (Sigma-Aldrich, 420220) until the following day. All cell lines were tested for mycoplasma contamination regularly with the PCR Mycoplasma Detection Set (TaKaRa, 6601) and maintained until passage 20. The pluripotency for each cell line was confirmed with an antibody against OCT4 (rabbit, 1:500, Abcam, ab19857) before each differentiation.

### Staining of iPSCs

In order to assess the pluripotency and proliferation of all iPSCs used in this study, three consecutive passages per cell line were cultured on coverslips, fixed and stained for OCT4 (rabbit, 1:500, Abcam, ab19857), Ki67 (rabbit, 1:400, Cell Signaling Technology, 9661S) and cCas3 (rabbit, 1:600, Merck, AB9260). The cells were fixed with 4% PFA in PBS (Roth, 1105.1) for 15 min and carefully washed twice with 1× PBS. Prior to staining, the cells were permeabilized with 0.5% Triton X-100 for 10 min at room temperature. After washing the cells with 1× PBS for 5 min, they were incubated in blocking buffer, consisting of 10% normal donkey serum (Abcam, ab7475) in PBS, for 1 h at room temperature. The primary antibodies were diluted in blocking buffer and administered to the coverslips overnight at 4°C. The cells were washed three times with 1× PBS for 5 min and incubated with the secondary antibodies in blocking buffer for 1 h at room temperature. After two washes with 1× PBS for 5 min, the cells were counterstained with DAPI (1:5000; Thermo Fisher Scientific, D1306) in PBS. Finally, the cells were washed once in 1× PBS and mounted on slides using ProLong Gold. For all cell lines and conditions, coverslips were imaged at 20× magnification.

### EdU incorporation

To quantify iPSC proliferation, three passages per cell line were treated with 10 µM EdU (Thermo Fisher Scientific, C10338) 3-4 days after the last passage for 1 and 4 h. After the incubation with EdU, cells were fixed and click chemistry was performed as advised by the manufacturer. The EdU signal was labeled with the provided Alexa Fluor 555 dye. Nuclear staining was performed with DAPI. Coverslips were imaged at 20× magnification on a confocal microscope, keeping laser settings identical for all cell lines and conditions.

### Quantification of immunohistochemistry and click chemistry on iPSCs

In order to quantify possible differences in the expression of markers for pluripotency, proliferation and apoptosis and in the number of cells positive for EdU, we stained and imaged respective samples in one experiment. All samples were imaged with the same laser intensity settings on a confocal microscope. Raw image files were further processed in FIJI (Schindelin et al., 2012). Here, we used the watershed algorithm on the DAPI channel to identify individual nuclei. These were then registered as ROIs and counted. To analyze the number of stained cells, thresholding the respective channel of interest was performed with identical parameters for each channel and all samples according to the negative staining control. ROIs demonstrating a signal for the channel of interest were then measured and counted. This allowed us to analyze the percentage of cells positive for the marker of interest within the total population of cells. Finally, statistical analysis and plotting were performed in GraphPad Prism.

### Generation of cerebellar organoids

Cerebellar organoids were generated as previously described (Silva et al., 2020) with some alterations: 80-90% confluent iPSCs were dissociated into single cells using accutase (Merck, A6964) and ∼4500 cells were seeded per well of 96-well V-bottom low-adhesion plates (S-Bio, MS-9096VZ) in E8 Flex medium supplemented with 10 μM Y-27632 (Cayman Chemical, 10005583). Once the aggregates reached a diameter of 250 μm, the medium was changed to growth factor-free chemically defined medium, supplemented with 50 ng/ml FGF2 and 10 μM SB-431542 (Tocris, 1614). At D7 of differentiation, FGF2 and SB-431542 were reduced to 33.3 ng/ml and 6.67 μM, respectively. At D14, the medium was supplemented with 100 ng/ml FGF19 (PeproTech, 100-32). The medium was changed to neurobasal medium (Gibco, 21103049) at D21, supplemented with 300 ng/ml SDF-1 (PeproTech, 300-28A) from D28 to D34. From D35 onwards, the medium was changed to complete BrainPhys (STEMCELL Technologies, 5793), supplemented with 10 μg/ml BDNF (PeproTech, 450-02), 100 μg/ml GDNF (PeproTech, 450-10), 100 mg/ml dbcAMP (PeproTech, 1698950) and 250 mM ascorbic acid (Tocris, 4055).

### Generation of cortical organoids

Cortical organoids were generated as previously described (Pasca et al., 2015) with only minor alterations. In brief, 80-90% confluent iPSCs were dissociated into single cells using accutase and ∼9000 cells were seeded per well of 96-well V-bottom low-adhesion plates in E8 Flex medium supplemented with 10 μM Y-27632 (Cayman Chemical, 10005583). The medium was changed to neural induction medium (Essential 6, Thermo Fisher Scientific, A151640), supplemented with 2.5 μM dorsomorphin (Tocris, 3093), 10 μM SB-431542 and 2.5 μM XAV-939 (Tocris, 3748) the next day. Neural induction medium was changed every other day and replaced with neural maintenance medium (NM) (Neurobasal-A, Gibco, 10888-022) containing 1× B27 supplement without Vitamin A (Thermo Fisher Scientific, 12587010), 1× GlutaMAX and 1× penicillin/streptomycin at D6. NM medium was supplemented with 20 ng/ml EGF (Merck, GF144) and 20 ng/ml FGF2 from D6 to D24 and with 20 ng/ml BDNF and 20 ng/ml NT-3 (PeproTech, 450-03) from D25 to D43. NM medium was changed every other day and not supplemented after D43.

### Size measurements

To investigate the size of organoids, brightfield images of the organoids were taken at D0, D10, D20, D30, D50, D70 and D90 of differentiation with an EVOS cell imaging system (Thermo Fisher Scientific). These images were analyzed using a published macro (Ivanov et al., 2014) for FIJI (Schindelin et al., 2012). The data were further analyzed with Excel, and Graph Pad Prism was used to plot data.

### Fixation, cryosections and immunohistochemistry

Organoids were fixed at the respective time points in 4% PFA in PBS for 45-60 min at room temperature (Lancaster and Knoblich, 2014). The organoids were washed three times for 15 min with 1× PBS and then incubated in 30% sucrose (Sigma-Aldrich, S7903) in PBS solution at 4°C until they sunk to the bottom of the dish. The organoids were embedded in a 1:1 v/v mixture of 30% sucrose in PBS and optimal cutting temperature (OCT) compound (Sakura, 4583) and sectioned on Superfrost Plus slides (R. Langenbrinck GmbH, 03-0060) with a cryostat at 20 µm (Leica). The slides were stored at −80°C.

For immunohistochemistry, slides were thawed for 15 min at room temperature and the embedding solution was rinsed off with PBS. Antigen retrieval was achieved by immersing the slides in 10 mM citric acid buffer (pH 6.0) and boiling for 20 min in a microwave. A hydrophobic pen (PAP pen, Abcam, ab2601) was used to circle the sections to prevent the blocking solution from spilling during incubation. Permeabilization and blocking were performed with 1% Triton X-100, 0.2% gelatin (Sigma-Aldrich, G1890) and 10% normal donkey serum in PBS for 1 h at room temperature. Primary antibodies were diluted in permeabilization and blocking solution and applied to the sections overnight at 4°C. Subsequently, the slides were rinsed with PBS three times for 15 min, then secondary antibodies were diluted in permeabilization and blocking solution and applied for 3 h at room temperature. Details of primary and secondary antibodies can be found in Tables S4 and S5. The sections were rinsed in PBS three times for 15 min and nuclei were stained with DAPI (1:5000) diluted in PBS for 4 min. The sections were then rinsed in PBS and mounted using ProLong Gold.

### Quantification of SOX2^+^ zones

To quantify SOX2^+^ area within individual organoids, we stained and imaged respective samples in one experiment. The laser intensity of the confocal microscope was adjusted according to the negative staining control. All samples were imaged with the same laser intensity settings. Raw image files were further processed in FIJI (Schindelin et al., 2012). To analyze the area of SOX2– zones, these regions were manually selected and measured. For each organoid and timepoint, all SOX2^+^ zones were quantified using the ‘measure’ tool in FIJI. The area of the SOX2^+^ zones was then normalized to the total DAPI^+^ area of the organoid. To measure the thickness of the SOX2^+^ zones, the outer and inner circumference was measured and the difference was calculated by subtracting the radius of the inner circumference from the radius of the outer circumference. Finally, statistical analysis and plotting were performed in GraphPad Prism.

### Quantification of cCas3 over SOX2 staining

To quantify cCas3^+^ area within ROIs in individual organoids, we stained and imaged respective samples in one experiment. The laser intensity of the confocal microscope was adjusted according to the negative staining control. All samples were imaged with the same laser intensity settings. Raw image files were further processed in FIJI (Schindelin et al., 2012). To analyze the area of stained cells, thresholding for cCas3 and SOX2 channels was performed with identical parameters for each channel and all samples. First, the area of cCas3-positively stained regions was quantified using the ‘measure’ tool in FIJI. Then, the cCas3^+^ area was normalized to the SOX2^+^ area of the respective image. Finally, statistical analysis and plotting were performed in GraphPad Prism.

### Quantification of Ki67^+^ cells over SOX2^+^ cells

To quantify Ki67^+^/SOX2^+^ cells within ROIs in individual organoids, we stained and imaged the respective samples in one experiment. The laser intensity of the confocal microscope was adjusted according to the negative staining control. All samples were imaged with the same laser intensity settings. Raw image files were further processed in FIJI (Schindelin et al., 2012). Thresholding was adjusted to the respective channel and, for each channel, identical parameters were used for all samples. Watershed algorithm was used on the thresholded SOX2 channel to identify ROIs. ROIs were saved in ROI manager and results and transferred to the thresholded Ki67 channel. The numbers of Ki67^+^ cells within these ROIs were counted and saved in the results file (Fig. S5). The results were exported to Excel to calculate percentages, and statistical analysis and plotting were performed in GraphPad Prism.

### Statistical analysis

Details of specific statistical comparisons are listed in the relevant figure legends. Two-tailed unpaired *t*-tests were performed without correction for multiple comparisons. An overview of the replicates is shown in Table S3. No formal comparison of variances was performed between experimental groups. No statistical methods were used to predetermine sample size.

## Supplementary Material

10.1242/dmm.050740_sup1 Supplementary information

Table S1. Clinical features of PCH2a probands.

Table S2. Linear regression model of regionalized neural organoid growth.

Table S3. Overview of experimental replicates.

Data S1. Karyotype reports of generated iPSCs.
