# Supplementary material for: Human organoid model of pontocerebellar hypoplasia 2a recapitulates brain region-specific size differences
Source: Dis Model Mech. 2024 Jul 22;17(7):dmm050740. doi: 10.1242/dmm.050740 (PMC11552497; doi:10.1242/dmm.050740)
Supplement: Supplementary information [file dmm-17-050740-s1.pdf]

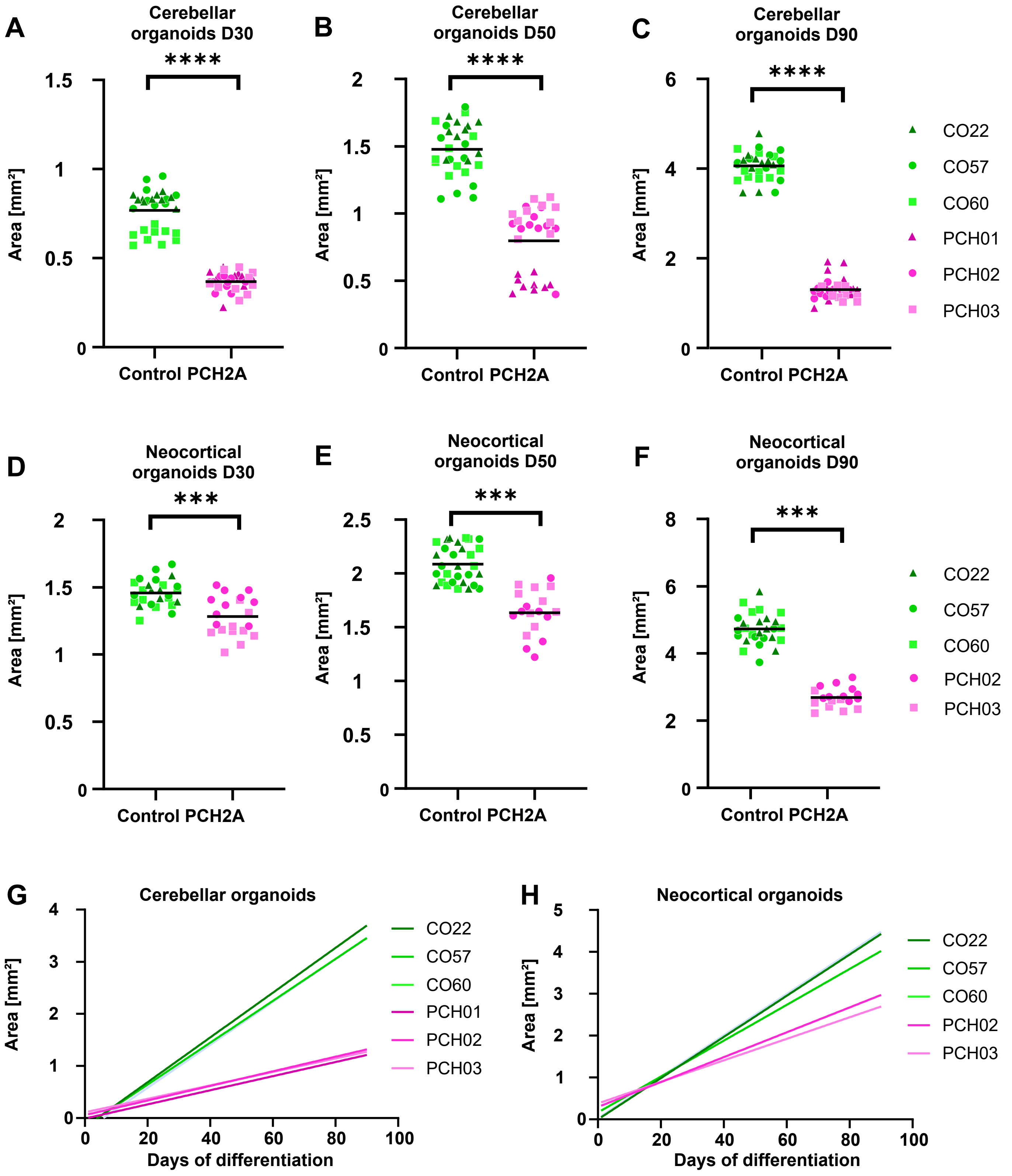

Fig. S1. Size differences of cerebellar and neocortical organoids at different stages of differentiation. Quantification of the area of cerebellar (A-C) and neocortical (D-F) control and PCH2a organoid areas at D30, D50 and D90 of differentiation. Sizes of organoids differ significantly between PCH2a and control organoids. (****, $p < 0.0001$, *** $p<0.001$ unpaired t-test with Welch's correction assuming unequal SDs). n>8 organoids per cell line, time point and differentiation. (G) Linear regression model for growth curves of cerebellar organoids. (H) Linear regression model for growth curves of neocortical organoids.

Disease Models & Mechanisms • Supplementary information

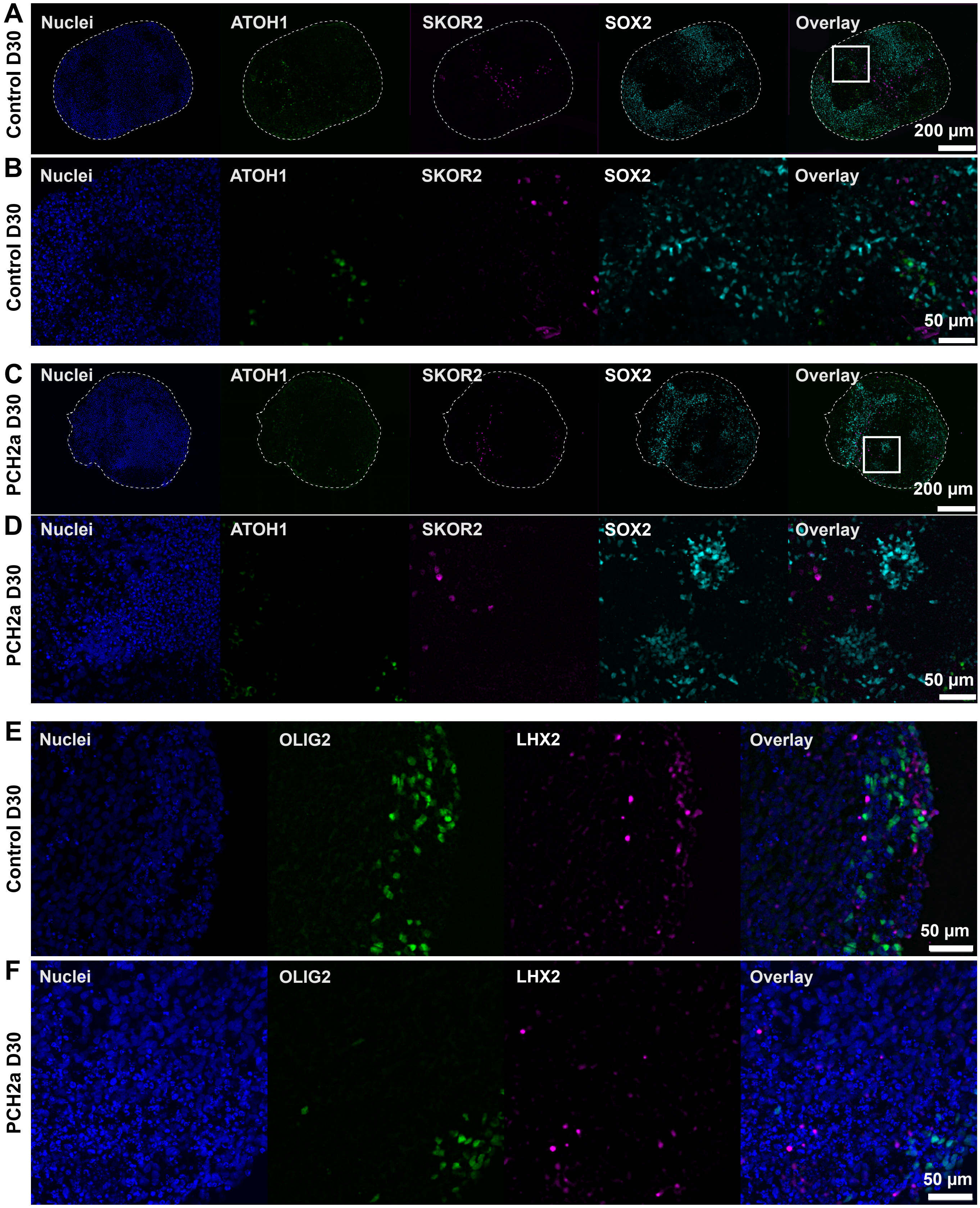

Disease Models & Mechanisms • Supplementary information

Fig. S2. PCH2a and control cerebellar organoids show differentiation into cerebellar lineage. (A-D) ATOH1 (green) expression demonstrates the presence of rhombic lip derived granule cell precursors while SKOR2 (magenta) is marker for young Purkinje cells derived from the cerebellar VZ, both markers are present in control (A, B) and PCH2a (C, D) cerebellar organoids (representative images CO57 P18, PCH03 P19). Dotted lines indicate the outline of organoids, white squares (A,C) indicate the region of the zoom-in (B, D). (E, F) Staining for early Purkinje marker OLIG2 (green) and LHX2 (magenta), early marker for nuclear transitory zone cells in Day 30 control (E) and PCH2a (F) cerebellar organoids (representative images CO60 P19, PCH01 P19). Nuclei are stained with DAPI.

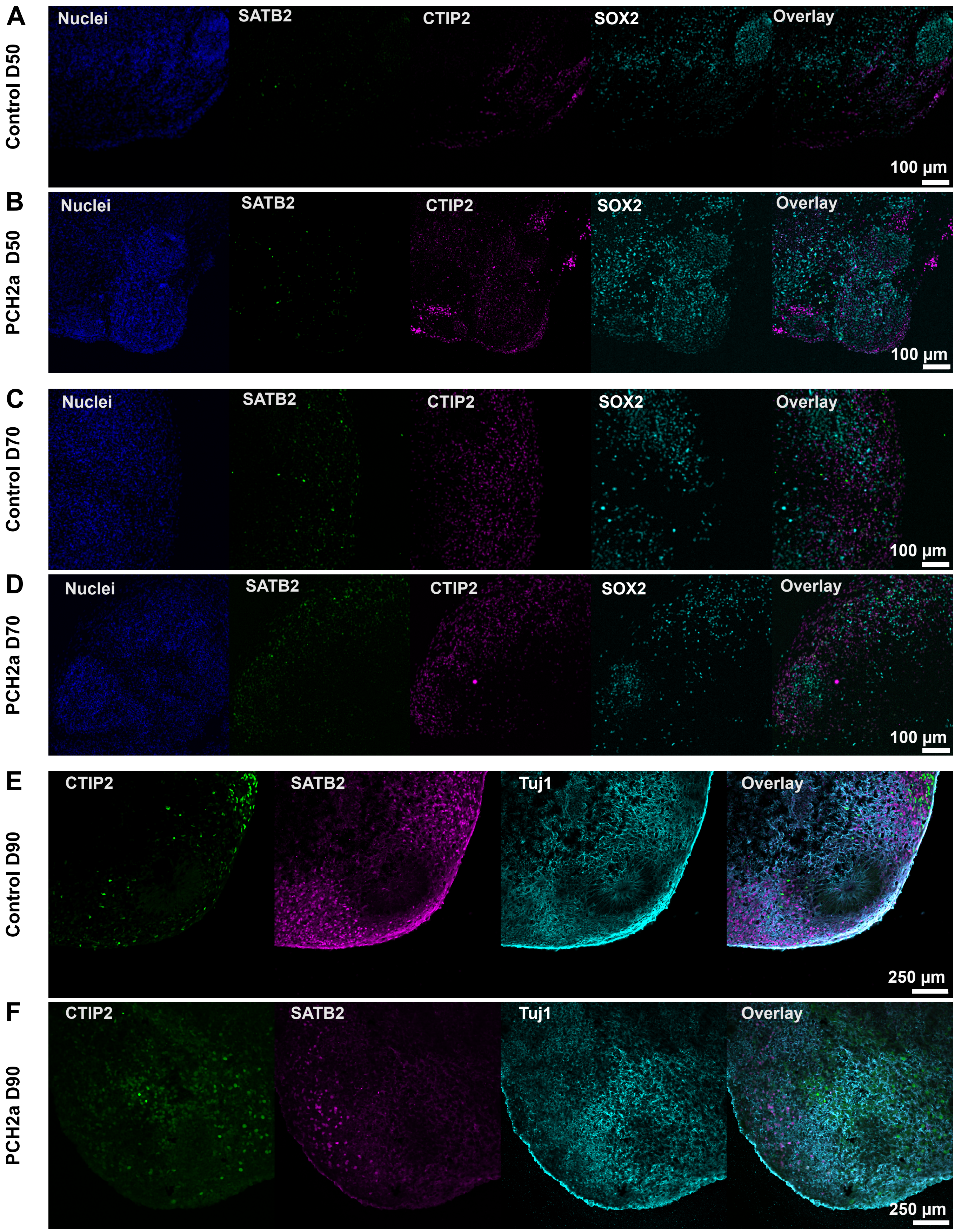
A
Control D50
Nuclei
SATB2
CTIP2
SOX2
Overlay
100 μm
B
PCH2a D50
Nuclei
SATB2
CTIP2
SOX2
Overlay
100 μm
C
Control D70
Nuclei
SATB2
CTIP2
SOX2
Overlay
100 μm
D
PCH2a D70
Nuclei
SATB2
CTIP2
SOX2
Overlay
100 μm
E
Control D90
CTIP2
SATB2
Tuj1
Overlay
250 μm
F
PCH2a D90
CTIP2
SATB2
Tuj1
Overlay
250 μm

Fig. S3. PCH2a and control neocortical organoids express layer-specific neuronal markers. (A-F) Epifluorescent microscopy images of immunohistochemistry on neocortical organoid sections. Control (A) and PCH2a (B) neocortical organoids express early-born deep-layer neuronal marker CTIP2 (magenta) at D50 of differentiation (representative images CO22 P19, PCH03 P19). (C, D, E, F) Control (C) and PCH2a (D) neocortical organoids show expression of CTIP2 as well upper layer neuronal marker SATB2 (green) at D70 (C, D) (representative images CO22 P19, PCH02 P17) and D90 (E, F) (representative images CO57 P18, PCH03 P19) of differentiation (CTIP2 green, SATB2 magenta). Nuclei are stained with DAPI.

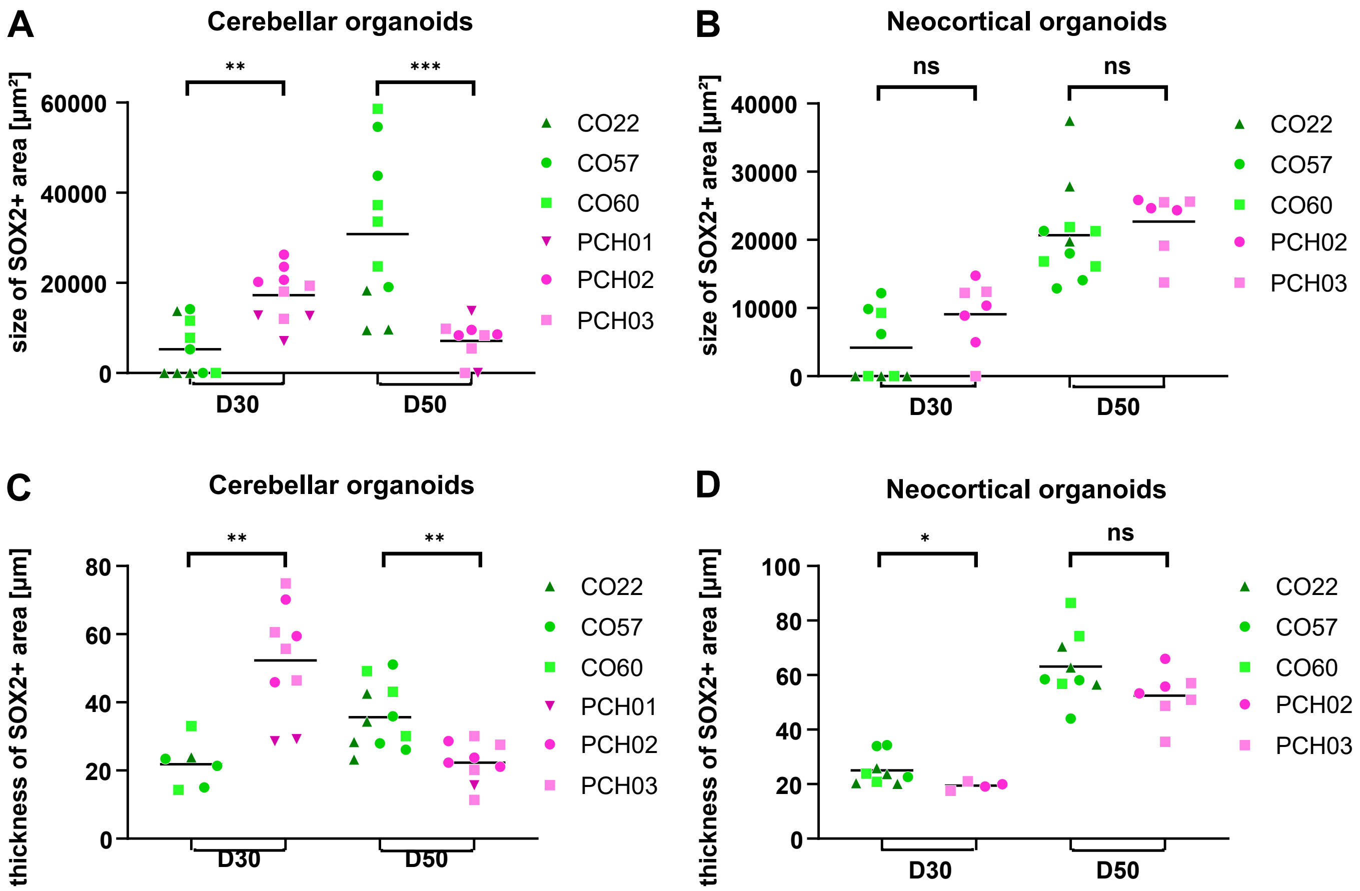

Fig. S4. Morphology of SOX2+ structures differs in PCH2a and control organoids. (A) SOX2+ structures are significantly bigger in PCH2a organoids at D30 of differentiation. At D50 control organoids show significantly bigger SOX2+ structures. (B) SOX2+ structures do not show difference in size at D30 and D50 of differentiation in neocortical organoids. (C) PCH2a cerebellar organoids show thicker SOX2+ structures at D30 of differentiation while SOX2+ structures are thicker in control organoids at D50. (D) Thickness of SOX2+ structures is lower in PCH2a neocortical organoids at D30 of differentiation. There is no difference in thickness of these structures at D50 of differentiation in neocortical organoids ($p>0.05$ for ns and $p<0.05$ for *, $p<0.01$ for**, $p<0.001$ for ***unpaired t-test with Welch's correction assuming unequal SDs).

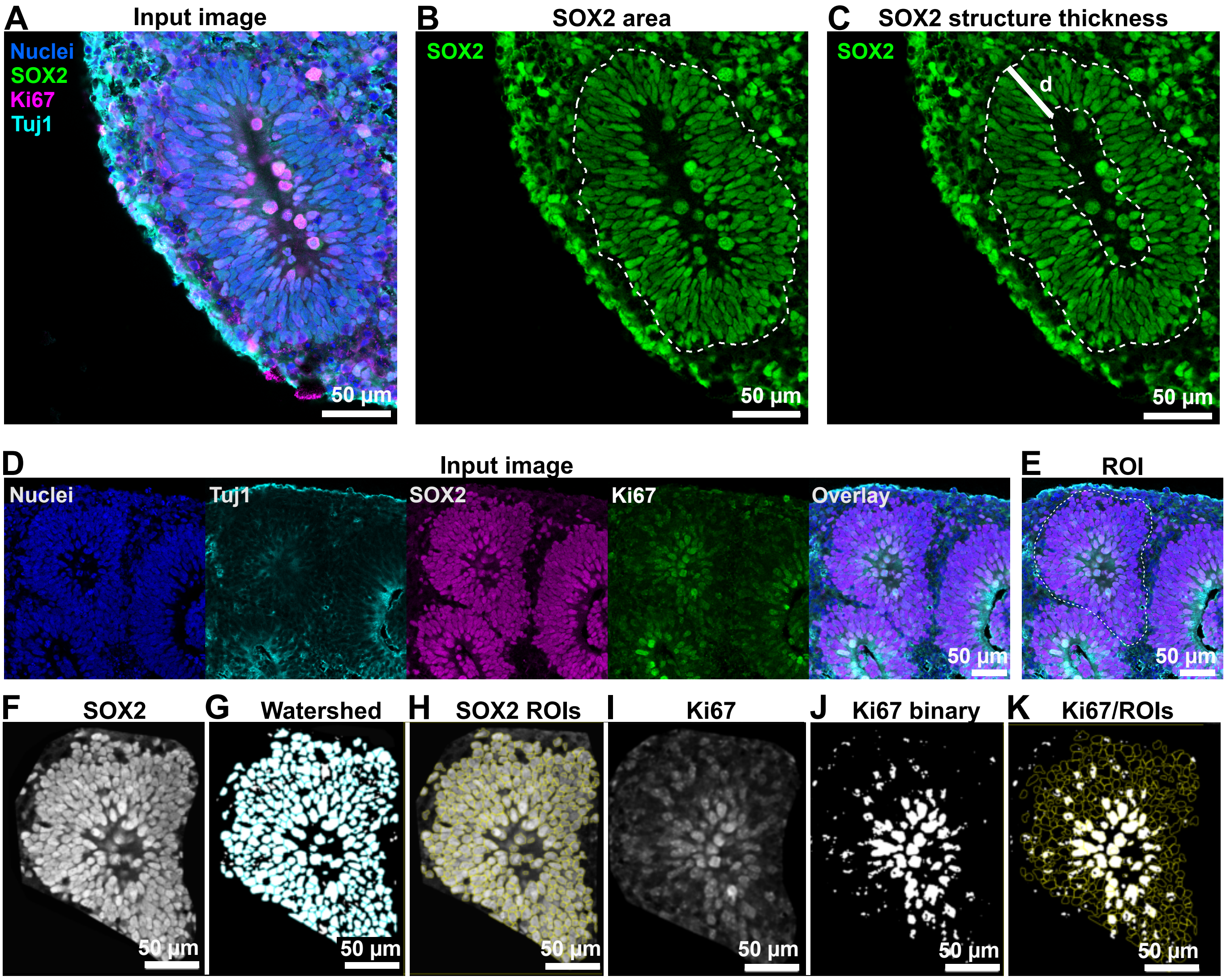

Fig. S5. Overview of methods for image analysis for SOX2+ rosettes. (A) Overlay of input image for size and thickness analysis of SOX2+ rosettes. (B) SOX2 channel of input image. White dotted line indicates outline of rosette used for size analysis of rosette structures. (C) SOX2 channel of input image. Outer dotted line indicates outline of SOX2+ rosette, while inner dotted structures indicates boarder to lumen. Thickness (d) of SOX2+ rosettes was calculated by subtracting the inner border from the circumference resulting in the mean thickness of the whole rosette structure. Images in A-C show a PCH2a cerebellar organoid at D30 of differentiation also shown in Fig. 6E. (D) Individual channels and overlay of input image for quantification of Ki67+/SOX2+ cells. (E) White dotted lines indicate region of interest (ROI) for quantification of Ki67+/SOX2+ cells. (F) Input SOX2 channel. (G) Watershed algorithm on thresholded SOX2 image. (H) Overlay of ROIs resulting from watershed algorithm on input image. (I) Ki67 input image. (J) Thresholded Ki67 image. (K) Overlay of SOX2 ROIs on thresholded Ki67 image. All SOX2 ROIs with Ki67 signal were counted as Ki67+/SOX2+ cells. Nuclei are stained with DAPI.

Disease Models & Mechanisms • Supplementary information

Table S1. Clinical features of PCH2a probands.

Available for download at
https://journals.biologists.com/dmm/article-lookup/doi/10.1242/dmm.050740#supplementary-data

Table S2. Linear regression model of regionalized neural organoid growth.

Available for download at
https://journals.biologists.com/dmm/article-lookup/doi/10.1242/dmm.050740#supplementary-data

Table S3. Overview of experimental replicates.

Available for download at
https://journals.biologists.com/dmm/article-lookup/doi/10.1242/dmm.050740#supplementary-data

Table S4. Primary Antibodies

Antibody	species	vendor	cat number	batch number	dilution
ATOH1	mouse	Merck	WH0000474M1	J8071-1B12	1:500
BAHRL1	rabbit	Atlas Antibodies	HPA004809	7306	1:500
Calbindin	mouse	Merck	C9848	125953	1:500
cCas3	rabbit	CST	9661S	47	1:400
CTIP2	rat	Abcam	ab18465	OR3427932,2	1:500
Ki67	rabbit	Merck	AB9260	41423360	1:600
Kirrel2	rabbit	Atlas Antibodies	HPA071587	R101296	1:500
LHX2	goat	Merck	SAB2500593	8573P1	1:500
Map2	rabbit	Abcam	ab32454	GR3453941-2	1:500
OCT4	rabbit	Abcam	ab19857	GR3284176-1	1:500
Olig2	rabbit	Merck	AB9610	3857662	1:500
SATB2	rabbit	Abcam	ab34735	GR3262034-2	1:500
SKOR2	rabbit	Atlas Antibodies	HPA046206	B96020	1:500
SOX2	goat	R&D Systems	AF2018	KOY0622061	1:500
TSEN54	rabbit	Invitrogen	PA5-101939	WL34558767	1:500
Tuj1	mouse	Atlas Antibodies	AMAb91394	3024	1:500

Table S5. Secondary antibodies

Host species	Target species	Fluorophore	Provider	Cat.no
donkey	goat	AF555	Abcam	ab150130
donkey	goat	AF647	Abcam	A21447
donkey	mouse	AF568	Abcam	ab175472
donkey	mouse	AF647	Abcam	A31571
donkey	rabbit	AF488	Abcam	A21206
donkey	rabbit	AF546	Abcam	A10040
donkey	rabbit	AF647	Abcam	A31573
donkey	sheep	AF488	Abcam	A11015
donkey	sheep	AF647	Abcam	A21448

Data S1. Karyotype reports of generated iPSCs.

Available for download at
https://journals.biologists.com/dmm/article-lookup/doi/10.1242/dmm.050740#supplementary-data

Disease Models & Mechanisms • Supplementary information
