# Supplementary material for: Human organoid model of pontocerebellar hypoplasia 2a recapitulates brain region-specific size differences
Source: Dis Model Mech. 2024 Jul 22;17(7):dmm050740. doi: 10.1242/dmm.050740 (PMC11552497; doi:10.1242/dmm.050740)
Supplement: Data S1. Karyotype reports of generated iPSCs. [file dmm-17-050740-DataS1.pdf]

# Karyotype Report

Customer sample ID: iPS-CO-22_2.1
Internal sample ID: DE04SCRHIHD100022
Date of receipt: 2020-02-26

Gender
Stated: Unknown
Chr. X derived: Male

Genotype identity with: DESCRHIH00023

## Karyotyping

Technology used: Illumina BeadArray

Product: HumanOmni2.5Exome-8 BeadChip v1.4
Manifest file: HumanOmni2-5Exome-8v1-4_A1.bpm
Cluster file: HumanOmni2-5Exome-8v1-4_A1.egt

Chip barcode and segment: 203730650093 R02C01

Batch ID and 96 well position: WG6911135-MSA6 B05

Call rate: 0,9976248

Typing
Scanner: Illumina iScan, S/N: N263
Site of processing: Life&Brain GENOMICS, Bonn, Germany
Manufacturer: Illumina, Inc., San Diego, United States of America
Date of scan: 2020-03-31-1456

Genotype Analysis
Genome Studio: GenomeStudio V2.0.4
Genotyping module: Ver. 2.0.4

Copy Number Analysis
Algorithm applied: CNV-Partition
Version: 3.2.0
Software producer: Illumina, Inc., San Diego, United States of America

### Noteworthy findings

No larger chromosomal aberrations to be reported.

## Control Dashboard

| Sample_ID / Sentrix_Label | Category | Control (BeadType) | Section 1 X | Section 1 Y | State |
|---|---|---|---|---|---|
| DESCRHIH00022 / 203730650093_R02C01 | Staining | DNP (High) (27630314) | 20239 | 110 | OK/OK |
| DESCRHIH00022 / 203730650093_R02C01 | Staining | DNP (Bgnd) (29619375) | 261 | 211 | OK/OK |
| DESCRHIH00022 / 203730650093_R02C01 | Staining | Biotin (High) (41666334) | 287 | 9696 | OK/OK |
| DESCRHIH00022 / 203730650093_R02C01 | Staining | Biotin (Bgnd) (34648333) | 220 | 84 | OK/OK |
| DESCRHIH00022 / 203730650093_R02C01 | Extension | Extension (A) (17616306) | 17544 | 294 | Notable/OK |
| DESCRHIH00022 / 203730650093_R02C01 | Extension | Extension (T) (14607337) | 19510 | 257 | Notable/OK |
| DESCRHIH00022 / 203730650093_R02C01 | Extension | Extension (C) (12613307) | 731 | 10601 | OK/OK |
| DESCRHIH00022 / 203730650093_R02C01 | Extension | Extension (G) (11603365) | 821 | 9764 | OK/Notable |
| DESCRHIH00022 / 203730650093_R02C01 | Target Removal | Target Removal (31623323) | 486 | 206 | OK/OK |
| DESCRHIH00022 / 203730650093_R02C01 | Hybridization | Hyb (High) (19612319) | 1167 | 10813 | OK/Notable |
| DESCRHIH00022 / 203730650093_R02C01 | Hybridization | Hyb (Medium) (20636378) | 349 | 6851 | OK/OK |
| DESCRHIH00022 / 203730650093_R02C01 | Hybridization | Hyb (Low) (23617335) | 982 | 2204 | OK/OK |
| DESCRHIH00022 / 203730650093_R02C01 | Stringency | String (PM) (32629312) | 12362 | 284 | Notable/OK |
| DESCRHIH00022 / 203730650093_R02C01 | Stringency | String (MM) (33668307) | 2315 | 182 | OK/OK |
| DESCRHIH00022 / 203730650093_R02C01 | Non-Specific Binding | NSB (Bgnd) (26619332) | 240 | 190 | OK/OK |
| DESCRHIH00022 / 203730650093_R02C01 | Non-Specific Binding | NSB (Bgnd) (27624356) | 223 | 186 | OK/OK |
| DESCRHIH00022 / 203730650093_R02C01 | Non-Specific Binding | NSB (Bgnd) (25617343) | 241 | 192 | OK/OK |
| DESCRHIH00022 / 203730650093_R02C01 | Non-Specific Binding | NSB (Bgnd) (24616350) | 264 | 190 | OK/OK |
| DESCRHIH00022 / 203730650093_R02C01 | Non-Polymorphic | NP (A) (34633358) | 6289 | 247 | OK/OK |
| DESCRHIH00022 / 203730650093_R02C01 | Non-Polymorphic | NP (T) (16648324) | 8394 | 246 | OK/OK |
| DESCRHIH00022 / 203730650093_R02C01 | Non-Polymorphic | NP (C) (43641328) | 448 | 5688 | OK/Notable |
| DESCRHIH00022 / 203730650093_R02C01 | Non-Polymorphic | NP (G) (13642359) | 472 | 5285 | OK/Notable |
| DESCRHIH00022 / 203730650093_R02C01 | Restoration | Restore (28637363) | 273 | 212 | OK/OK |

All „Notable“ tagged probes are within specs. The karyogram can be evaluated.

# Karyogram

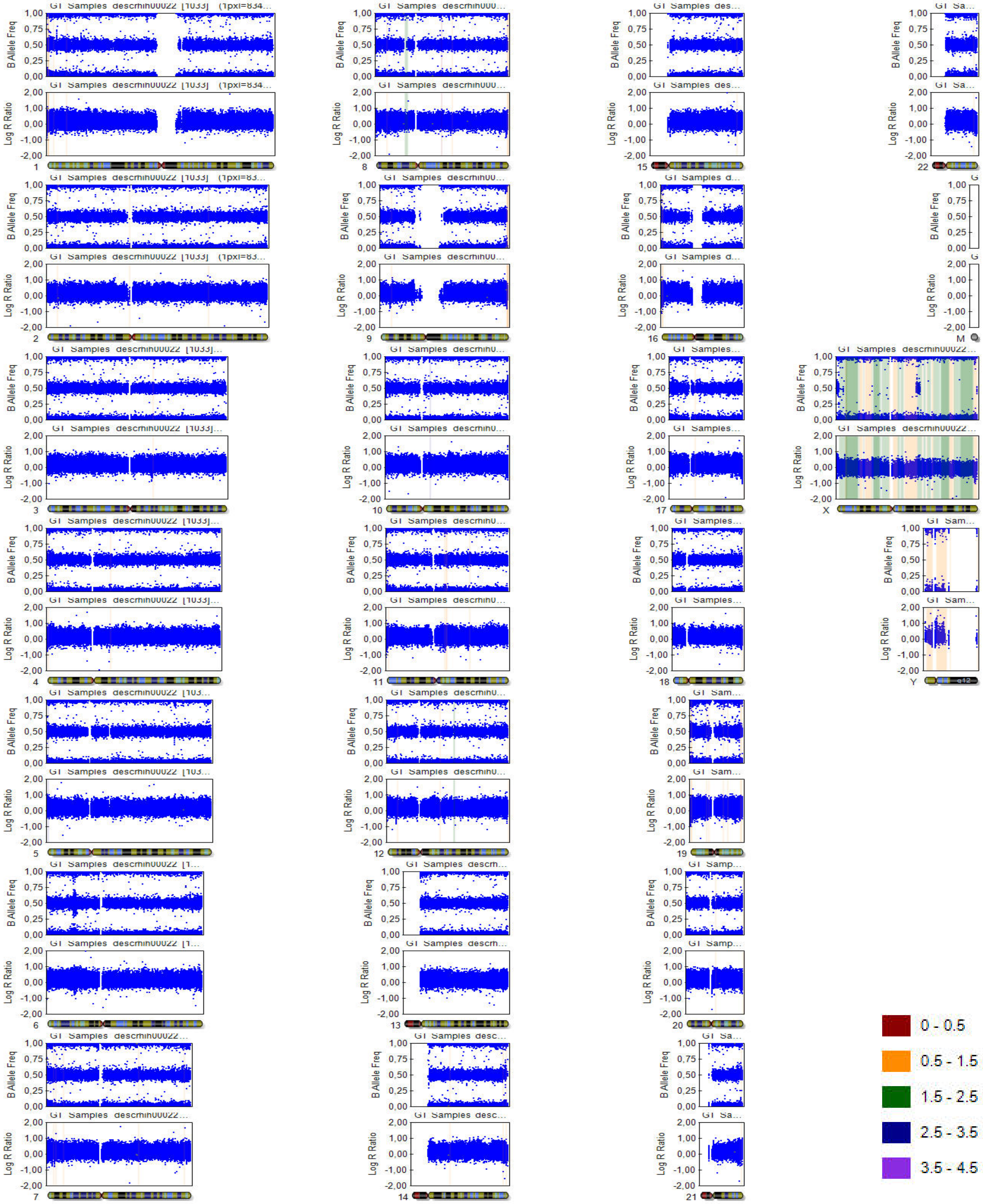

## Copy Number Analysis

Copy number events will be reported if larger than 350'000 base pairs, 1Mbp for loss of heterozygosity regions.
Database of Genomic Variants comparison: Number stated represent population based copy number variants that span the reported event completely.

Copy number analysis

Algorithm applied: CNV-Partition
Version: 3.2
Software producer: Illumina, Inc., San Diego, United States of America

| Sample ID | Chr | Start | End | Type | Length | Marker count | CN Confidence | DGV Comparison |
|---|---|---|---|---|---|---|---|---|
| DESCRHIH00022 | 8 | 31781189 | 35324420 | 2 | 3543231 | 2334 | 455,1505 | |
| DESCRHIH00022 | 12 | 72825139 | 74758965 | 2 | 1933826 | 1162 | 235,608 | |

# Karyotype Report

Customer sample ID: 27_iPS-CO-57cl.B.1
Internal sample ID: DE86SCRDZND100143
Date of receipt: 26.07.2018

Gender
Stated: Unknown
Chr. X derived: Male

Genotype identity with: 28_F-CO-57-2018-07-26 / DESCRDZN00144

## Karyotyping

Technology used: Illumina BeadArray

Product: HumanOmni2.5Exome-8 BeadChip v1.3
Manifest file: HumanOmni2-5Exome-8v1-3_A1.bpm
Cluster file: HumanOmni2-5Exome-8v1-3_A1.egt

Chip barcode and segment: 201364830139 R03C01

Batch ID and 96 well position: WG6906661-MSA6 C04

Call rate: 0,9981163

Typing
Scanner: Illumina iScan, S/N: N234
Site of processing: Life&Brain GENOMICS, Bonn, Germany
Manufacturer: Illumina, Inc., San Diego, United States of America
Date of scan: 2018-08-01-2310

Genotype Analysis
Genome Studio: GenomeStudio V2.0.2
Genotyping module: Ver. 2.0.2

Copy Number Analysis
Algorithm applied: CNV-Partition
Version: 3.2
Software producer: Illumina, Inc., San Diego, United States of America

### Noteworthy findings

No larger chromosomal aberrations to be reported.

## Control Dashboard

| Sample_ID / Sentrix_Label | Category | Control (BeadType) | Section 1 X | Section 1 Y | State |
|---|---|---|---|---|---|
| DESCRDZN00143 / 201364830139_R03C01 | Staining | DNP (High) (27630314) | 27305 | 137 | OK/OK |
| DESCRDZN00143 / 201364830139_R03C01 | Staining | DNP (Bgnd) (29619375) | 387 | 223 | OK/OK |
| DESCRDZN00143 / 201364830139_R03C01 | Staining | Biotin (High) (41666334) | 486 | 12740 | OK/OK |
| DESCRDZN00143 / 201364830139_R03C01 | Staining | Biotin (Bgnd) (34648333) | 374 | 100 | OK/OK |
| DESCRDZN00143 / 201364830139_R03C01 | Extension | Extension (A) (17616306) | 27672 | 396 | OK/OK |
| DESCRDZN00143 / 201364830139_R03C01 | Extension | Extension (T) (14607337) | 30291 | 315 | OK/OK |
| DESCRDZN00143 / 201364830139_R03C01 | Extension | Extension (C) (12613307) | 1063 | 13549 | OK/OK |
| DESCRDZN00143 / 201364830139_R03C01 | Extension | Extension (G) (11603365) | 1345 | 13010 | OK/OK |
| DESCRDZN00143 / 201364830139_R03C01 | Target Removal | Target Removal (31623323) | 677 | 179 | OK/OK |
| DESCRDZN00143 / 201364830139_R03C01 | Hybridization | Hyb (High) (19612319) | 1329 | 12828 | OK/OK |
| DESCRDZN00143 / 201364830139_R03C01 | Hybridization | Hyb (Medium) (20636378) | 550 | 7772 | OK/OK |
| DESCRDZN00143 / 201364830139_R03C01 | Hybridization | Hyb (Low) (23617335) | 1251 | 2588 | OK/OK |
| DESCRDZN00143 / 201364830139_R03C01 | Stringency | String (PM) (32629312) | 17938 | 361 | OK/OK |
| DESCRDZN00143 / 201364830139_R03C01 | Stringency | String (MM) (33668307) | 4743 | 270 | OK/OK |
| DESCRDZN00143 / 201364830139_R03C01 | Non-Specific Binding | NSB (Bgnd) (26619332) | 365 | 166 | OK/OK |
| DESCRDZN00143 / 201364830139_R03C01 | Non-Specific Binding | NSB (Bgnd) (27624356) | 350 | 164 | OK/OK |
| DESCRDZN00143 / 201364830139_R03C01 | Non-Specific Binding | NSB (Bgnd) (25617343) | 347 | 185 | OK/OK |
| DESCRDZN00143 / 201364830139_R03C01 | Non-Specific Binding | NSB (Bgnd) (24616350) | 380 | 156 | OK/OK |
| DESCRDZN00143 / 201364830139_R03C01 | Non-Polymorphic | NP (A) (34633358) | 9101 | 296 | OK/OK |
| DESCRDZN00143 / 201364830139_R03C01 | Non-Polymorphic | NP (T) (16648324) | 11388 | 240 | OK/OK |
| DESCRDZN00143 / 201364830139_R03C01 | Non-Polymorphic | NP (C) (43641328) | 619 | 6573 | OK/OK |
| DESCRDZN00143 / 201364830139_R03C01 | Non-Polymorphic | NP (G) (13642359) | 517 | 5944 | OK/Notable |
| DESCRDZN00143 / 201364830139_R03C01 | Restoration | Restore (28637363) | 371 | 197 | OK/OK |

All „Notable“ tagged probes are within specs. The karyogram can be evaluated.

# Karyogram

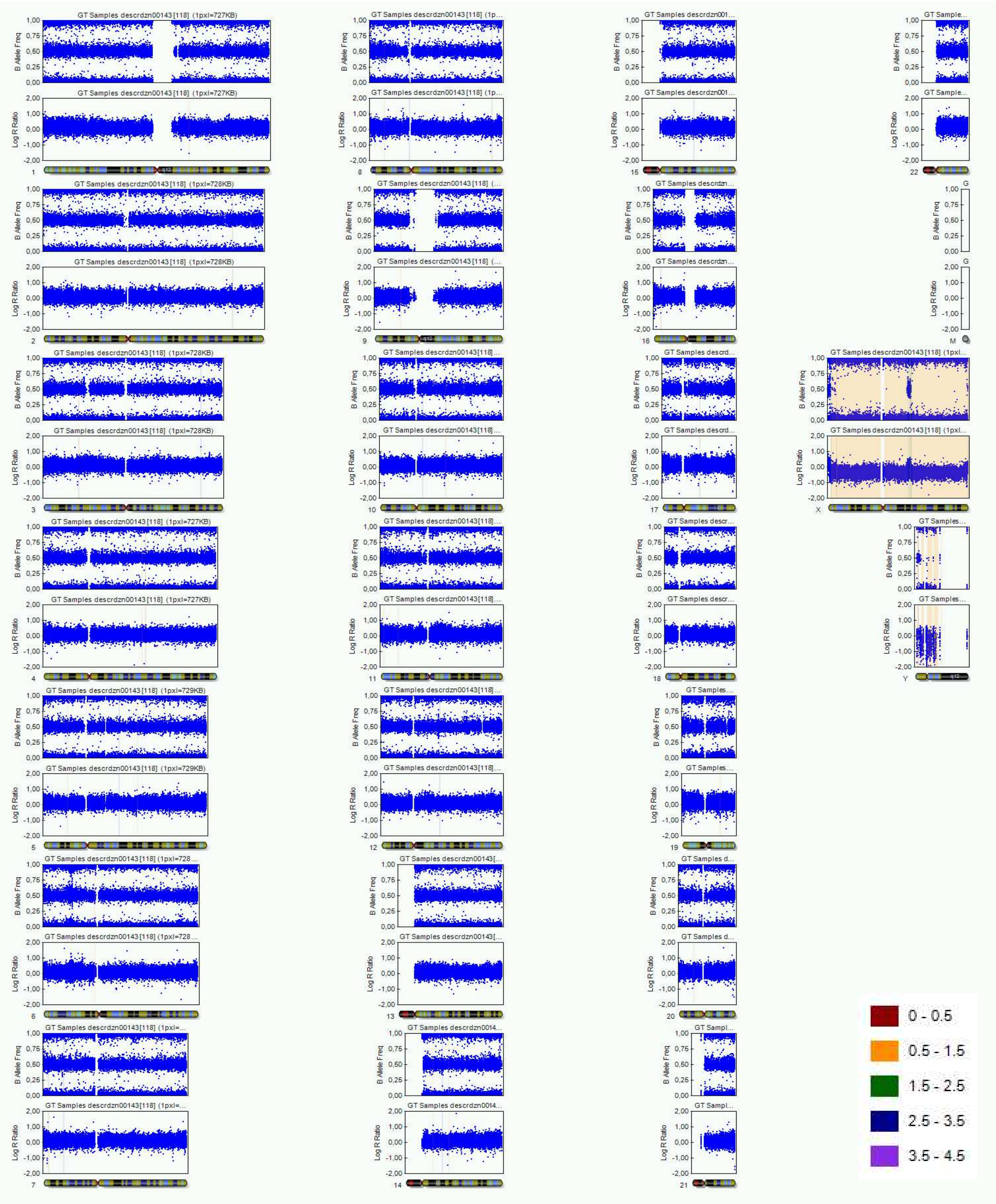

## Copy Number Analysis

Copy number events will be reported if larger than 350'000 base pairs, 1Mbp for loss of heterozygosity regions.
Database of Genomic Variants comparison: Number stated represent population based copy number variants that span the reported event completely.

Copy number analysis
Algorithm applied: CNV-Partition
Version: 3.2
Software producer: Illumina, Inc., San Diego, United States of America

| Sample ID | Chr | Start | End | Type | Length | Marker count | CN Confidence | DGV Comparison |
|---|---|---|---|---|---|---|---|---|
| DESCRDZN00143 | 8 | 43057452 | 43806224 | 3 | 748772 | 361 | 427,1952 | |

# Karyotype Report

Customer sample ID: iPS-CO-60_4.3
Internal sample ID: DE47SCRHIHD100024
Date of receipt: 2020-02-26

Gender
Stated: Unknown
Chr. X derived: Male

Genotype identity with: DESCRHIH00025

## Karyotyping

Technology used: Illumina BeadArray

Product: HumanOmni2.5Exome-8 BeadChip v1.4
Manifest file: HumanOmni2-5Exome-8v1-4_A1.bpm
Cluster file: HumanOmni2-5Exome-8v1-4_A1.egt

Chip barcode and segment: 203730650093 R04C01

Batch ID and 96 well position: WG6911135-MSA6 D05

Call rate: 0,997606

Typing
Scanner: Illumina iScan, S/N: N263
Site of processing: Life&Brain GENOMICS, Bonn, Germany
Manufacturer: Illumina, Inc., San Diego, United States of America
Date of scan: 2020-03-31-1503

Genotype Analysis
Genome Studio: GenomeStudio V2.0.4
Genotyping module: Ver. 2.0.4

Copy Number Analysis
Algorithm applied: CNV-Partition
Version: 3.2.0
Software producer: Illumina, Inc., San Diego, United States of America

### Noteworthy findings

No larger chromosomal aberrations to be reported.

## Control Dashboard

| Sample_ID / Sentrix_Label | Category | Control (BeadType) | Section 1 X | Section 1 Y | State |
|---|---|---|---|---|---|
| DESCRHIH00024 / 203730650093_R04C01 | Staining | DNP (High) (27630314) | 21958 | 137 | OK/OK |
| DESCRHIH00024 / 203730650093_R04C01 | Staining | DNP (Bgnd) (29619375) | 344 | 203 | OK/OK |
| DESCRHIH00024 / 203730650093_R04C01 | Staining | Biotin (High) (41666334) | 321 | 12082 | OK/OK |
| DESCRHIH00024 / 203730650093_R04C01 | Staining | Biotin (Bgnd) (34648333) | 247 | 110 | OK/OK |
| DESCRHIH00024 / 203730650093_R04C01 | Extension | Extension (A) (17616306) | 20971 | 373 | OK/OK |
| DESCRHIH00024 / 203730650093_R04C01 | Extension | Extension (T) (14607337) | 23411 | 283 | OK/OK |
| DESCRHIH00024 / 203730650093_R04C01 | Extension | Extension (C) (12613307) | 878 | 12869 | OK/OK |
| DESCRHIH00024 / 203730650093_R04C01 | Extension | Extension (G) (11603365) | 959 | 12222 | OK/OK |
| DESCRHIH00024 / 203730650093_R04C01 | Target Removal | Target Removal (31623323) | 598 | 252 | OK/OK |
| DESCRHIH00024 / 203730650093_R04C01 | Hybridization | Hyb (High) (19612319) | 1366 | 13100 | OK/OK |
| DESCRHIH00024 / 203730650093_R04C01 | Hybridization | Hyb (Medium) (20636378) | 384 | 8349 | OK/OK |
| DESCRHIH00024 / 203730650093_R04C01 | Hybridization | Hyb (Low) (23617335) | 1176 | 2737 | OK/OK |
| DESCRHIH00024 / 203730650093_R04C01 | Stringency | String (PM) (32629312) | 14996 | 379 | OK/OK |
| DESCRHIH00024 / 203730650093_R04C01 | Stringency | String (MM) (33668307) | 3141 | 269 | OK/OK |
| DESCRHIH00024 / 203730650093_R04C01 | Non-Specific Binding | NSB (Bgnd) (26619332) | 313 | 201 | OK/OK |
| DESCRHIH00024 / 203730650093_R04C01 | Non-Specific Binding | NSB (Bgnd) (27624356) | 324 | 197 | OK/OK |
| DESCRHIH00024 / 203730650093_R04C01 | Non-Specific Binding | NSB (Bgnd) (25617343) | 299 | 228 | OK/OK |
| DESCRHIH00024 / 203730650093_R04C01 | Non-Specific Binding | NSB (Bgnd) (24616350) | 296 | 226 | OK/OK |
| DESCRHIH00024 / 203730650093_R04C01 | Non-Polymorphic | NP (A) (34633358) | 7804 | 266 | OK/OK |
| DESCRHIH00024 / 203730650093_R04C01 | Non-Polymorphic | NP (T) (16648324) | 9916 | 297 | OK/OK |
| DESCRHIH00024 / 203730650093_R04C01 | Non-Polymorphic | NP (C) (43641328) | 522 | 7149 | OK/OK |
| DESCRHIH00024 / 203730650093_R04C01 | Non-Polymorphic | NP (G) (13642359) | 537 | 6188 | OK/OK |
| DESCRHIH00024 / 203730650093_R04C01 | Restoration | Restore (28637363) | 363 | 206 | OK/OK |

All probes are within specs. The karyogram can be evaluated.

life&brain

# Karyogram

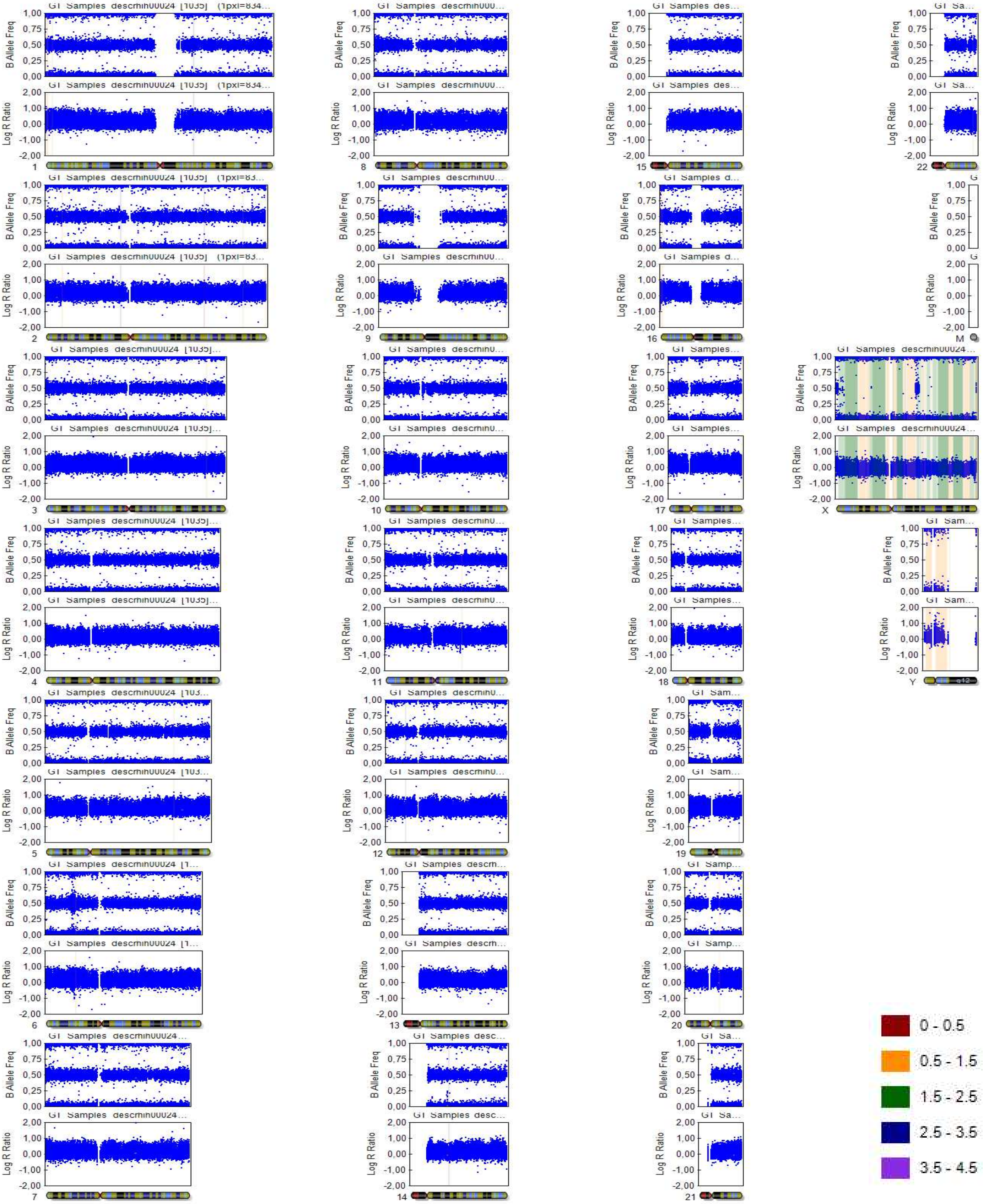

# Copy Number Analysis

Copy number events will be reported if larger than 350'000 base pairs, 1Mbp for loss of heterozygosity regions.
Database of Genomic Variants comparison: Number stated represent population based copy number variants that span the reported event completely.

Copy number analysis
Algorithm applied: CNV-Partition
Version: 3.2
Software producer: Illumina, Inc., San Diego, United States of America

| Sample ID | Chr | Start | End | Type | Length | Marker count | CN Confidence | DGV Comparison |
|---|---|---|---|---|---|---|---|---|
| DESCRHIH00024 | 12 | 20522796 | 21680693 | 2 | 1157897 | 1493 | 342,3974 | |
| DESCRHIH00024 | 23 | 2700157 | 24993887 | 2 | 22293730 | 11958 | 1481,305 | |
| DESCRHIH00024 | 23 | 25000842 | 33074340 | 1 | 8073498 | 4676 | 676,9131 | |
| DESCRHIH00024 | 23 | 33112111 | 37029338 | 1 | 3917227 | 1767 | 289,3273 | |
| DESCRHIH00024 | 23 | 37033484 | 54733713 | 2 | 17700229 | 7686 | 1490,719 | |
| DESCRHIH00024 | 23 | 54736227 | 58563509 | 1 | 3827282 | 990 | 70,9988 | |
| DESCRHIH00024 | 23 | 61686465 | 62573853 | 1 | 887388 | 185 | 146,1671 | |
| DESCRHIH00024 | 23 | 63400883 | 67085251 | 1 | 3684368 | 976 | 271,2258 | CNV-Gain: 1, OTHER-Inversion: 2 |
| DESCRHIH00024 | 23 | 67097144 | 74027649 | 2 | 6930505 | 2957 | 2351,022 | |
| DESCRHIH00024 | 23 | 74031648 | 76230687 | 1 | 2199039 | 618 | 66,9812 | |
| DESCRHIH00024 | 23 | 76237943 | 77787620 | 2 | 1549677 | 293 | 678,5046 | |
| DESCRHIH00024 | 23 | 77804801 | 88440097 | 1 | 10635296 | 4446 | 1318,529 | |
| DESCRHIH00024 | 23 | 88442047 | 92374313 | 2 | 3932266 | 1160 | 248,2036 | |
| DESCRHIH00024 | 23 | 92547980 | 94025957 | 2 | 1477977 | 831 | 1801,044 | |
| DESCRHIH00024 | 23 | 94027807 | 95538643 | 1 | 1510836 | 738 | 313,9972 | |
| DESCRHIH00024 | 23 | 95544051 | 97068109 | 2 | 1524058 | 763 | 1833,992 | |
| DESCRHIH00024 | 23 | 97074770 | 99554768 | 1 | 2479998 | 903 | 165,7551 | |
| DESCRHIH00024 | 23 | 99557421 | 104463794 | 2 | 4906373 | 1423 | 3124,86 | |

| | | | | | | | | |
|---|---|---|---|---|---|---|---|---|
| DESCRHIH00024 | 23 | 104464069 | 106109204 | 1 | 1645135 | 428 | 164,2652 | |
| DESCRHIH00024 | 23 | 106112751 | 111616869 | 2 | 5504118 | 1301 | 963,7 | |
| DESCRHIH00024 | 23 | 111650324 | 123584863 | 2 | 11934539 | 2763 | 1477,011 | |
| DESCRHIH00024 | 23 | 123588245 | 128397152 | 1 | 4808907 | 897 | 251,0483 | |
| DESCRHIH00024 | 23 | 128398819 | 140002446 | 2 | 11603627 | 2758 | 1564,544 | |
| DESCRHIH00024 | 23 | 140010139 | 147122830 | 1 | 7112691 | 1731 | 612,3635 | |
| DESCRHIH00024 | 23 | 147158718 | 152213311 | 2 | 5054593 | 1464 | 1059,053 | |
| DESCRHIH00024 | 23 | 152222116 | 153282028 | 1 | 1059912 | 385 | 427,3397 | |
| DESCRHIH00024 | 23 | 153698458 | 154916845 | 2 | 1218387 | 380 | 825,4448 | CNV-Gain: 1, CNV-Gain+Loss: 1 |
| DESCRHIH00024 | 24 | 2657176 | 10082095 | 1 | 7424919 | 728 | 3551,399 | |
| DESCRHIH00024 | 24 | 13133499 | 24891486 | 1 | 11757987 | 1499 | 7184,58 | |

# Karyotype Report

Customer sample ID: iPS-PCH-01_3.1_p11
Internal sample ID: DE34SCRDZND100206
Date of receipt: 2021-03-09

Gender
Stated: Unstated
Chr. X derived: Male

Genotype identity with: F-PCH-01_P3-2021-03-09 / DESCRDZN00202, IPS-PCH-01_2.1_P11-2021-03-09 / DESCRDZN00205

## Karyotyping

Technology used: Illumina BeadArray

Product: HumanOmni2.5Exome-8 BeadChip v1.5
Manifest file: InfiniumOmni2-5Exome-8v1-5_A1.bpm
Cluster file: InfiniumOmni2-5Exome-8v1-5_A1-Cluster-File.egt

Chip barcode and segment: 204372050103 R07C01

Batch ID and 96 well position: WG6917121-MSA6 G02

Call rate: 0,9986958

Typing
Scanner: Illumina iScan, S/N: N234
Site of processing: Life&Brain GENOMICS, Bonn, Germany
Manufacturer: Illumina, Inc., San Diego, United States of America
Date of scan: 2021-03-17-1200

Genotype Analysis
Genome Studio: GenomeStudio V2.0.5
Genotyping module: Ver. 2.0.5

Copy Number Analysis
Algorithm applied: CNV-Partition
Version: 3.2.0
Software producer: Illumina, Inc., San Diego, United States of America

### Noteworthy findings

No larger chromosomal aberrations to be reported.

## Control Dashboard

| Sample_ID / Sentrix_Label | Category | Control (BeadType) | Section 1 X | Section 1 Y | State |
|---|---|---|---|---|---|
| DESCRDZN00206 / 204372050103_R07C01 | Staining | DNP (High) (27630314) | 37630 | 369 | OK/OK |
| DESCRDZN00206 / 204372050103_R07C01 | Staining | DNP (Bgnd) (43603326) | 710 | 450 | Notable/ Notable |
| DESCRDZN00206 / 204372050103_R07C01 | Staining | Biotin (High) (41666334) | 792 | 20828 | OK/OK |
| DESCRDZN00206 / 204372050103_R07C01 | Staining | Biotin (Bgnd) (34648333) | 582 | 355 | Notable/OK |
| DESCRDZN00206 / 204372050103_R07C01 | Extension | Extension (A) (17616306) | 36574 | 466 | OK/OK |
| DESCRDZN00206 / 204372050103_R07C01 | Extension | Extension (T) (14607337) | 37520 | 410 | OK/OK |
| DESCRDZN00206 / 204372050103_R07C01 | Extension | Extension (C) (12613307) | 1845 | 21676 | OK/OK |
| DESCRDZN00206 / 204372050103_R07C01 | Extension | Extension (G) (11603365) | 2118 | 21803 | Notable/OK |
| DESCRDZN00206 / 204372050103_R07C01 | Target Removal | Target Removal (31623323) | 1220 | 256 | OK/OK |
| DESCRDZN00206 / 204372050103_R07C01 | Hybridization | Hyb (High) (19612319) | 1778 | 22924 | OK/OK |
| DESCRDZN00206 / 204372050103_R07C01 | Hybridization | Hyb (Medium) (20636378) | 771 | 15775 | OK/OK |
| DESCRDZN00206 / 204372050103_R07C01 | Hybridization | Hyb (Low) (23617335) | 1949 | 4464 | OK/OK |
| DESCRDZN00206 / 204372050103_R07C01 | Stringency | String (PM) (32629312) | 26788 | 516 | OK/OK |
| DESCRDZN00206 / 204372050103_R07C01 | Stringency | String (MM) (33668307) | 9148 | 380 | Notable/OK |
| DESCRDZN00206 / 204372050103_R07C01 | Non-Specific Binding | NSB (Bgnd) (28637363) | 528 | 370 | OK/OK |
| DESCRDZN00206 / 204372050103_R07C01 | Non-Specific Binding | NSB (Bgnd) (27624356) | 596 | 283 | OK/OK |
| DESCRDZN00206 / 204372050103_R07C01 | Non-Specific Binding | NSB (Bgnd) (25617343) | 1828 | 287 | OK/OK |
| DESCRDZN00206 / 204372050103_R07C01 | Non-Specific Binding | NSB (Bgnd) (24616350) | 558 | 1113 | OK/OK |
| DESCRDZN00206 / 204372050103_R07C01 | Non-Polymorphic | NP(A) (34633358) | 13152 | 391 | OK/OK |
| DESCRDZN00206 / 204372050103_R07C01 | Non-Polymorphic | NP(T) (16648324) | 19273 | 444 | OK/OK |
| DESCRDZN00206 / 204372050103_R07C01 | Non-Polymorphic | NP(C) (43641328) | 901 | 11699 | OK/OK |
| DESCRDZN00206 / 204372050103_R07C01 | Non-Polymorphic | NP(G) (13642359) | 932 | 11048 | OK/OK |

All „Notable“ tagged probes are within specs. The karyogram can be evaluated.

life&brain

## Karyogram

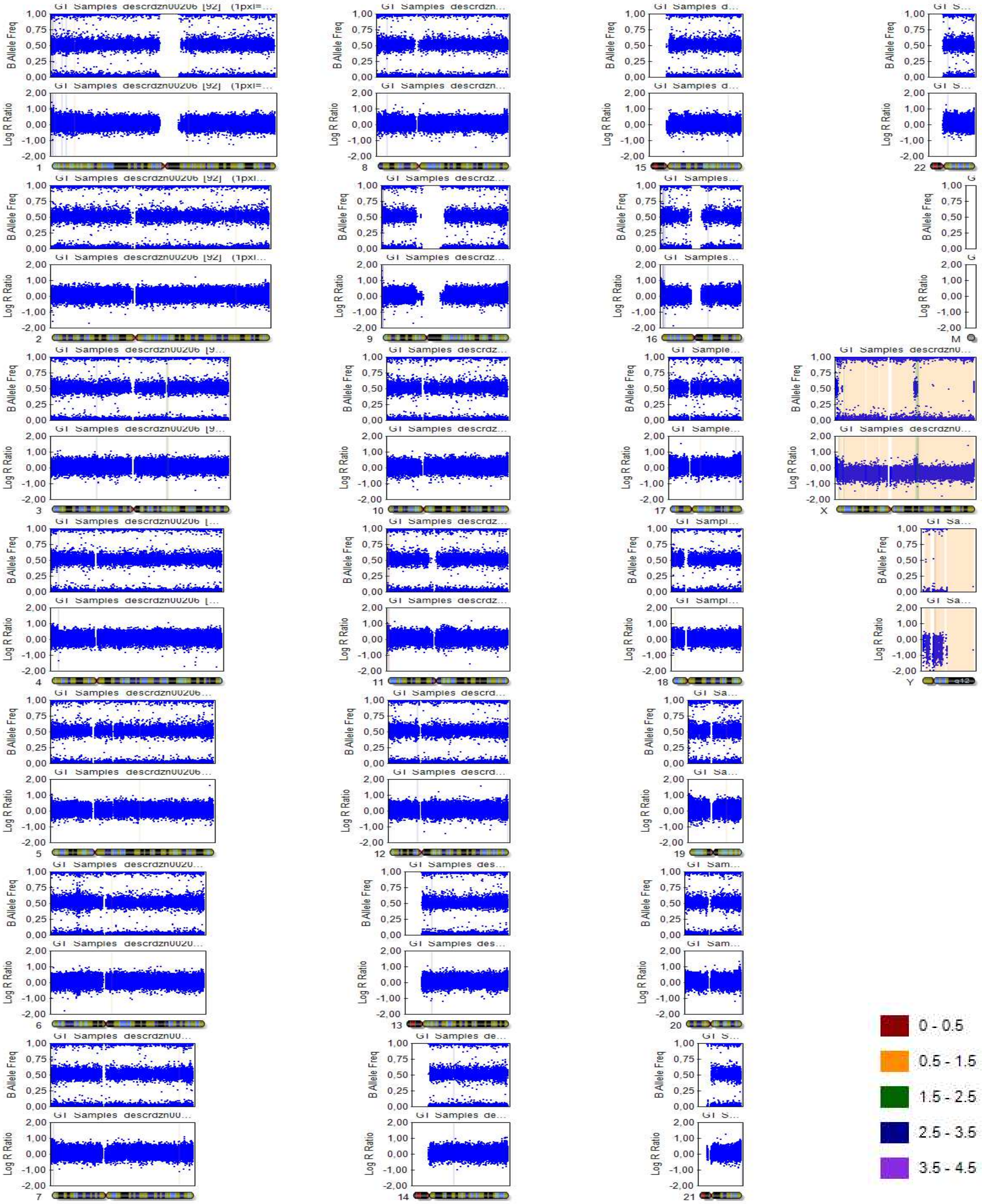

## Copy Number Analysis

Copy number events will be reported if larger than 350'000 base pairs, 1Mbp for loss of heterozygosity regions.
Database of Genomic Variants comparison: Number stated represent population based copy number variants that span the reported event completely.

Copy number analysis

Algorithm applied: CNV-Partition
Version: 3.2
Software producer: Illumina, Inc., San Diego, United States of America

| Sample ID | Chr | Start | End | Type | Length | Marker count | CN Confidence | DGV Comparison |
|---|---|---|---|---|---|---|---|---|
| DESCRDZN00206 | 3 | 127465894 | 129762545 | 2 | 2296651 | 1816 | 283,233 | |

# Karyotype Report

Customer sample ID: iPS-PCH-02_1.7_p10
Internal sample ID: DE07SCRDZND100207
Date of receipt: 2021-03-09

Gender
Stated: Unstated
Chr. X derived: Male

Genotype identity with: F-PCH-02_P5-2021-03-09 / DESCRDZN00203,
IPS-PCH-02_4.1_P11-2021-03-09 / DESCRDZN00208

## Karyotyping

Technology used: Illumina BeadArray

Product: HumanOmni2.5Exome-8 BeadChip v1.5
Manifest file: InfiniumOmni2-5Exome-8v1-5_A1.bpm
Cluster file: InfiniumOmni2-5Exome-8v1-5_A1-Cluster-File.egt

Chip barcode and segment: 204372050103 R08C01

Batch ID and 96 well position: WG6917121-MSA6 H02

Call rate: 0,9970004

Typing
Scanner: Illumina iScan, S/N: N234
Site of processing: Life&Brain GENOMICS, Bonn, Germany
Manufacturer: Illumina, Inc., San Diego, United States of America
Date of scan: 2021-03-17-1200

Genotype Analysis
Genome Studio: GenomeStudio V2.0.5
Genotyping module: Ver. 2.0.5

Copy Number Analysis
Algorithm applied: CNV-Partition
Version: 3.2.0
Software producer: Illumina, Inc., San Diego, United States of America

### Noteworthy findings

No larger chromosomal aberrations to be reported.

## Control Dashboard

| Sample_ID / Sentrix_Label | Category | Control (BeadType) | Section 1 X | Section 1 Y | State |
|---|---|---|---|---|---|
| DESCRDZN00207 / 204372050103_R08C01 | Staining | DNP (High) (27630314) | 43484 | 373 | OK/OK |
| DESCRDZN00207 / 204372050103_R08C01 | Staining | DNP (Bgnd) (43603326) | 813 | 392 | Notable/ Notable |
| DESCRDZN00207 / 204372050103_R08C01 | Staining | Biotin (High) (41666334) | 1013 | 24996 | Notable/OK |
| DESCRDZN00207 / 204372050103_R08C01 | Staining | Biotin (Bgnd) (34648333) | 743 | 362 | Notable/OK |
| DESCRDZN00207 / 204372050103_R08C01 | Extension | Extension (A) (17616306) | 39698 | 614 | OK/OK |
| DESCRDZN00207 / 204372050103_R08C01 | Extension | Extension (T) (14607337) | 40283 | 498 | OK/OK |
| DESCRDZN00207 / 204372050103_R08C01 | Extension | Extension (C) (12613307) | 1959 | 25122 | OK/OK |
| DESCRDZN00207 / 204372050103_R08C01 | Extension | Extension (G) (11603365) | 2321 | 25291 | Notable/OK |
| DESCRDZN00207 / 204372050103_R08C01 | Target Removal | Target Removal (31623323) | 1360 | 398 | OK/OK |
| DESCRDZN00207 / 204372050103_R08C01 | Hybridization | Hyb (High) (19612319) | 1643 | 25731 | OK/OK |
| DESCRDZN00207 / 204372050103_R08C01 | Hybridization | Hyb (Medium) (20636378) | 876 | 17961 | OK/OK |
| DESCRDZN00207 / 204372050103_R08C01 | Hybridization | Hyb (Low) (23617335) | 1879 | 4195 | OK/OK |
| DESCRDZN00207 / 204372050103_R08C01 | Stringency | String (PM) (32629312) | 28809 | 621 | OK/OK |
| DESCRDZN00207 / 204372050103_R08C01 | Stringency | String (MM) (33668307) | 6714 | 417 | Notable/OK |
| DESCRDZN00207 / 204372050103_R08C01 | Non-Specific Binding | NSB (Bgnd) (28637363) | 719 | 402 | Notable/OK |
| DESCRDZN00207 / 204372050103_R08C01 | Non-Specific Binding | NSB (Bgnd) (27624356) | 691 | 344 | OK/OK |
| DESCRDZN00207 / 204372050103_R08C01 | Non-Specific Binding | NSB (Bgnd) (25617343) | 661 | 380 | OK/OK |
| DESCRDZN00207 / 204372050103_R08C01 | Non-Specific Binding | NSB (Bgnd) (24616350) | 699 | 357 | OK/OK |
| DESCRDZN00207 / 204372050103_R08C01 | Non-Polymorphic | NP(A) (34633358) | 12123 | 492 | OK/OK |
| DESCRDZN00207 / 204372050103_R08C01 | Non-Polymorphic | NP(T) (16648324) | 19309 | 448 | OK/OK |
| DESCRDZN00207 / 204372050103_R08C01 | Non-Polymorphic | NP(C) (43641328) | 951 | 13059 | OK/OK |
| DESCRDZN00207 / 204372050103_R08C01 | Non-Polymorphic | NP(G) (13642359) | 961 | 12365 | OK/OK |

All „Notable“ tagged probes are within specs. The karyogram can be evaluated.

# Karyogram

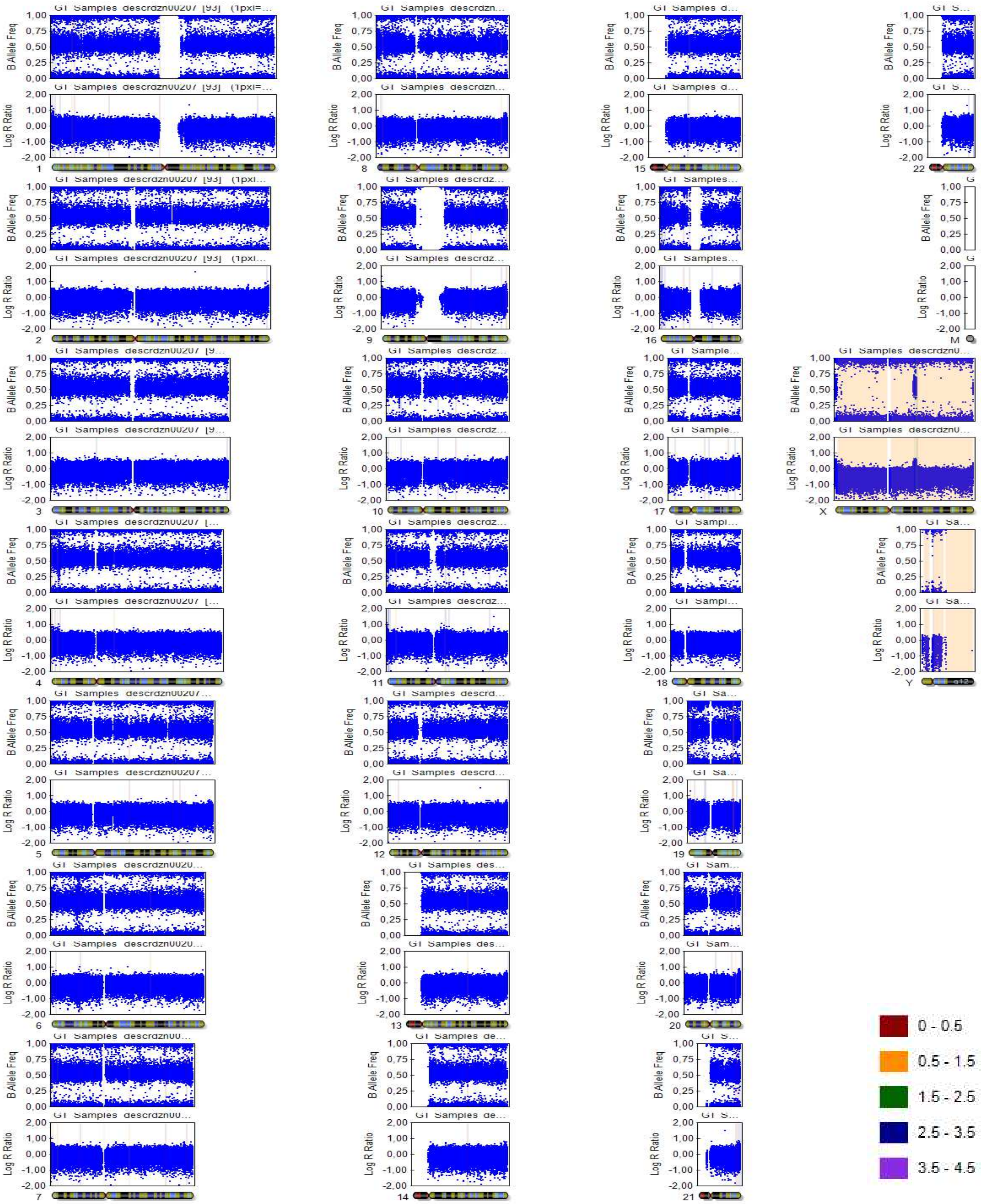

## Copy Number Analysis

Copy number events will be reported if larger than 350'000 base pairs, 1Mbp for loss of heterozygosity regions.
Database of Genomic Variants comparison: Number stated represent population based copy number variants that span the reported event completely.

Copy number analysis

Algorithm applied: CNV-Partition
Version: 3.2
Software producer: Illumina, Inc., San Diego, United States of America

| Sample ID | Chr | Start | End | Type | Length | Marker count | CN Confidence | DGV Comparison |
|---|---|---|---|---|---|---|---|---|
| DESCRDZN00207 | 4 | 9558683 | 10407505 | 3 | 848822 | 710 | 2740,706 | |

# Karyotype Report

Customer sample ID: iPS-PCH-03_4.2_p10
Internal sample ID: DE50SCRDZND100209
Date of receipt: 2021-03-09

Gender
Stated: Unstated
Chr. X derived: Male

Genotype identity with: F-PCH-03_P2-2021-03-09 / DESCRDZN00204,
IPS-PCH-03_6.1_P11-2021-03-09 / DESCRDZN00210

## Karyotyping

Technology used: Illumina BeadArray

Product: HumanOmni2.5Exome-8 BeadChip v1.5
Manifest file: InfiniumOmni2-5Exome-8v1-5_A1.bpm
Cluster file: InfiniumOmni2-5Exome-8v1-5_A1-Cluster-File.egt

Chip barcode and segment: 204372060010 R02C01

Batch ID and 96 well position: WG6917121-MSA6 B03

Call rate: 0,9982454

Typing
Scanner: Illumina iScan, S/N: N234
Site of processing: Life&Brain GENOMICS, Bonn, Germany
Manufacturer: Illumina, Inc., San Diego, United States of America
Date of scan: 2021-03-17-1200

Genotype Analysis
Genome Studio: GenomeStudio V2.0.5
Genotyping module: Ver. 2.0.5

Copy Number Analysis
Algorithm applied: CNV-Partition
Version: 3.2.0
Software producer: Illumina, Inc., San Diego, United States of America

**Noteworthy findings**

No larger chromosomal aberrations to be reported.

## Control Dashboard

| Sample_ID / Sentrix_Label | Category | Control (BeadType) | Section 1 X | Section 1 Y | State |
|---|---|---|---|---|---|
| DESCRDZN00209 / 204372060010_R02C01 | Staining | DNP (High) (27630314) | 30251 | 86 | OK/OK |
| DESCRDZN00209 / 204372060010_R02C01 | Staining | DNP (Bgnd) (43603326) | 339 | 156 | Notable/ Notable |
| DESCRDZN00209 / 204372060010_R02C01 | Staining | Biotin (High) (41666334) | 465 | 11921 | OK/OK |
| DESCRDZN00209 / 204372060010_R02C01 | Staining | Biotin (Bgnd) (34648333) | 363 | 141 | OK/OK |
| DESCRDZN00209 / 204372060010_R02C01 | Extension | Extension (A) (17616306) | 26250 | 248 | OK/OK |
| DESCRDZN00209 / 204372060010_R02C01 | Extension | Extension (T) (14607337) | 27511 | 254 | OK/OK |
| DESCRDZN00209 / 204372060010_R02C01 | Extension | Extension (C) (12613307) | 1074 | 12240 | OK/OK |
| DESCRDZN00209 / 204372060010_R02C01 | Extension | Extension (G) (11603365) | 1331 | 11312 | OK/OK |
| DESCRDZN00209 / 204372060010_R02C01 | Target Removal | Target Removal (31623323) | 643 | 156 | OK/OK |
| DESCRDZN00209 / 204372060010_R02C01 | Hybridization | Hyb (High) (19612319) | 1131 | 12010 | OK/OK |
| DESCRDZN00209 / 204372060010_R02C01 | Hybridization | Hyb (Medium) (20636378) | 414 | 8273 | OK/OK |
| DESCRDZN00209 / 204372060010_R02C01 | Hybridization | Hyb (Low) (23617335) | 998 | 2530 | OK/OK |
| DESCRDZN00209 / 204372060010_R02C01 | Stringency | String (PM) (32629312) | 18022 | 296 | OK/OK |
| DESCRDZN00209 / 204372060010_R02C01 | Stringency | String (MM) (33668307) | 5625 | 182 | OK/OK |
| DESCRDZN00209 / 204372060010_R02C01 | Non-Specific Binding | NSB (Bgnd) (28637363) | 358 | 156 | OK/OK |
| DESCRDZN00209 / 204372060010_R02C01 | Non-Specific Binding | NSB (Bgnd) (27624356) | 336 | 162 | OK/OK |
| DESCRDZN00209 / 204372060010_R02C01 | Non-Specific Binding | NSB (Bgnd) (25617343) | 347 | 184 | OK/OK |
| DESCRDZN00209 / 204372060010_R02C01 | Non-Specific Binding | NSB (Bgnd) (24616350) | 286 | 178 | OK/OK |
| DESCRDZN00209 / 204372060010_R02C01 | Non-Polymorphic | NP(A) (34633358) | 8841 | 256 | OK/OK |
| DESCRDZN00209 / 204372060010_R02C01 | Non-Polymorphic | NP(T) (16648324) | 12595 | 230 | OK/OK |
| DESCRDZN00209 / 204372060010_R02C01 | Non-Polymorphic | NP(C) (43641328) | 549 | 6457 | OK/OK |
| DESCRDZN00209 / 204372060010_R02C01 | Non-Polymorphic | NP(G) (13642359) | 644 | 6025 | OK/OK |

All „Notable“ tagged probes are within specs. The karyogram can be evaluated.

## Karyogram

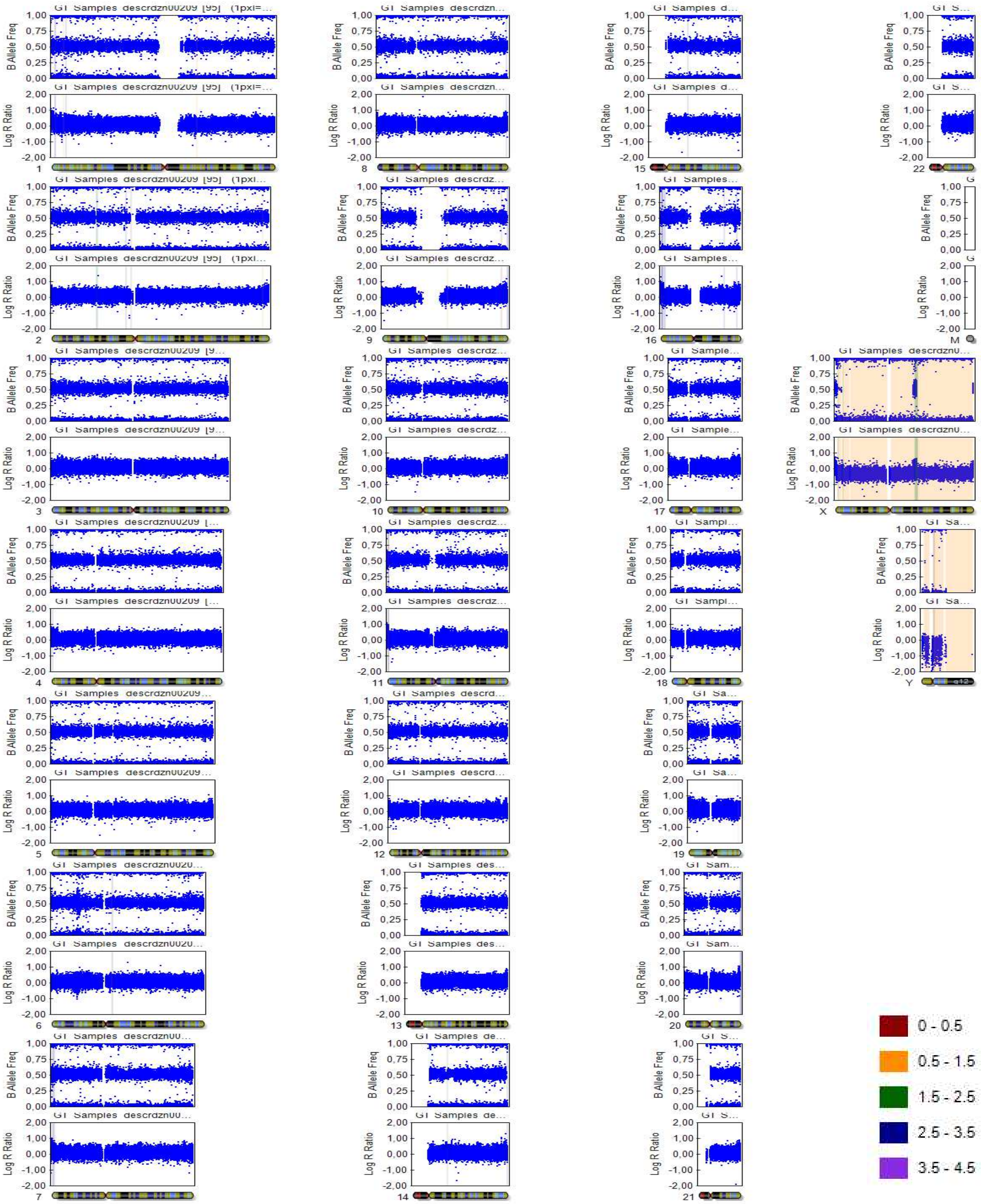

## Copy Number Analysis

Copy number events will be reported if larger than 350'000 base pairs, 1Mbp for loss of heterozygosity regions.
Database of Genomic Variants comparison: Number stated represent population based copy number variants that span the reported event completely.

Copy number analysis
Algorithm applied: CNV-Partition
Version: 3.2
Software producer: Illumina, Inc., San Diego, United States of America

| Sample ID | Chr | Start | End | Type | Length | Marker count | CN Confidence | DGV Comparison |
|---|---|---|---|---|---|---|---|---|
| DESCRDZN00209 | 2 | 49977093 | 52048553 | 2 | 2071460 | 1910 | 481,6987 | |
| DESCRDZN00209 | 14 | 38487404 | 40341185 | 2 | 1853781 | 1534 | 333,2639 | |
